# Beyond pCR Prediction: Subtype-Specific Artificial Intelligence for Treatment Tailoring in Breast Cancer Neoadjuvant Therapy

**DOI:** 10.3390/cancers18182947

**Published:** 2026-09-11

**Authors:** Junwen Zhou, Beibei Xi, Kehuan Yan, Linying Chen

**Affiliations:** 1 College of Computer and Data Science, Fuzhou University, Fuzhou 350108, China; 2501010020@fzu.edu.cn (J.Z.); 241010006@fzu.edu.cn (K.Y.); 2 Department of Pathology, The First Affiliated Hospital of Fujian Medical University, Fuzhou 350005, China; beibeixi@fjmu.edu.cn

**Keywords:** breast cancer, neoadjuvant therapy, pathologic complete response, artificial intelligence, clinical prediction models

## Abstract

Neoadjuvant therapy for breast cancer is planned by subtype: pathologic complete response (pCR) differs in frequency, meaning, and surrogate validity across HR+/HER2−, HER2+, and triple-negative breast cancer (TNBC). This review examines whether current evidence supports using artificial intelligence (AI) to move beyond predicting response under a fixed regimen to guiding systemic treatment tailoring. Here, tailoring means model-guided drug omission, switching, escalation, or de-escalation for an individual patient. We appraised 102 full-text studies of AI-based response prediction (2020–2026), organized by subtype and clinical decision, and graded each on an author-defined five-level clinical-readiness ladder (L1–L5) measuring validation and translational maturity rather than accuracy. Risk of bias was assessed with PROBAST and reporting against TRIPOD+AI. Readiness clustered low: 43 studies reached internal validation only (L1), 53 temporal or geographic external validation (L2), and 6 prospective observational validation (L3); none reached workflow integration (L4) or interventional evidence (L5). Most carried high overall risk of bias (89/102); external validation appeared in 52 (51%), fully reported decision-curve analysis in 44 (43%), and calibration in 19 (19%). Strategy comparison and individualized-treatment-effect analyses were essentially absent. Subtype-specific AI currently predicts response under fixed regimens with moderate-to-good discrimination; the evidence does not yet support changing an individual patient’s systemic treatment.

## Simple Summary

Breast cancer comprises biologically distinct subtypes rather than a single disease, and each is treated with a different pre-surgery strategy. Clinicians want to know early whether a patient is likely to respond, because that could guide safer treatment adjustment. Artificial intelligence has therefore been used to estimate response from routinely collected imaging, pathology slides, and laboratory data, using models that range from statistical scores to deep-learning systems and multimodal designs. This review finds such estimates are not interchangeable across subtypes and must be read in their original treatment context. Most models were tested only where they were built, few in independent patients, and fewer still for reliable probabilities or improved decisions. Current evidence is therefore not strong enough for AI to change an individual treatment or surgery decision safely. We map how mature the evidence is, how it differs across subtypes, and what is still required.

## 1. Introduction

Neoadjuvant therapy provides a window to observe breast cancer response before surgery and to inform subsequent treatment. Its purpose now extends beyond tumor shrinkage to subtype-specific decisions about systemic therapy and surgical extent. HR+/HER2−, HER2+, and triple-negative breast cancer (TNBC) differ in biology, treatment, and the meaning of response. A pooled response score can obscure these differences. This review asks whether artificial intelligence (AI) can move from predicting response to supporting treatment tailoring, and examines that question separately for each subtype.

Established treatment pathways show why the distinction matters. KATHERINE supported trastuzumab emtansine (T-DM1) for observed residual HER2+ disease [1]; KEYNOTE-522 demonstrated event-free-survival benefit from immunochemotherapy in TNBC [2]. Pathologic complete response (pCR) has different prognostic meaning across subtypes, with a weaker survival association in HR+/HER2− disease [3]. Residual cancer burden (RCB) and recurrence risk may therefore better match some clinical questions. Changing targets alone does not guarantee improvement: deep-learning radiomics failed to improve RCB prediction over simpler measures in the LIMA MRI cohort [4]. Histopathology-based recurrence and chemotherapy-benefit models broaden the questions under study [5]. Table 1 summarizes endpoint definitions and their clinical meaning.

AI approaches include radiomics, deep learning, digital pathology, and multimodal fusion, which combines inputs such as imaging, pathology, and clinical variables. Their predictions require three separate judgments. Discrimination measures how well a model ranks responders above non-responders, commonly summarized by the area under the receiver operating characteristic curve (AUC). Calibration measures agreement between predicted probabilities and observed outcomes. A calibrated 30% response probability means that approximately 30 of 100 comparable patients respond. Clinical utility asks whether acting on a prediction does more good than harm. Decision-curve analysis (DCA) estimates potential net benefit at specified thresholds against treating all or no patients; it does not demonstrate prospective clinical benefit. Appendix A (Table A1) provides the technical glossary.

Here, treatment tailoring means using a model to omit, switch, escalate, or de-escalate an individual patient’s systemic therapy. Surgical or axillary management, monitoring without a treatment change, and prognostic stratification are related applications with different evidentiary requirements. We identify these applications explicitly rather than equating them with systemic-treatment selection.

**Figure 1:**
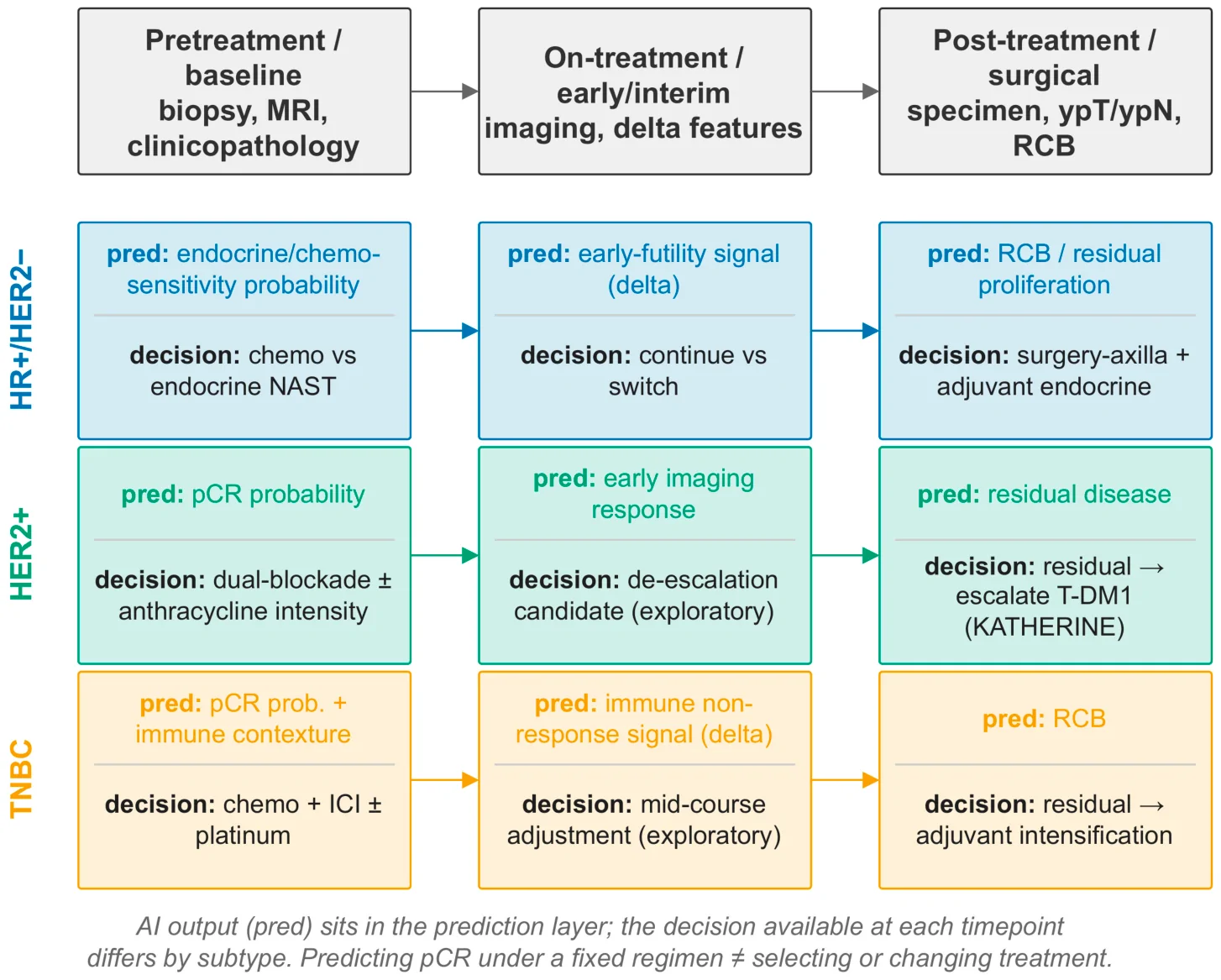
Clinical decision pathway across breast cancer subtypes in the neoadjuvant setting. AI output remains in the prediction layer; the decision available at each timepoint differs by subtype.

Predicting pCR under a fixed regimen is a prognostic claim. Selecting treatment requires evidence about alternatives, through strategy comparison, individualized treatment-effect (ITE) estimation, or prospective interventional testing. ITE denotes the difference a treatment would make for an individual compared with an alternative. We assess models against this distinction and their intended decision, with Figure 1 mapping the subtype-specific clinical pathway.

The clinical application must therefore be specified before interpreting a response score. The same output may support prognosis, motivate surgical research, or suggest an adaptive-treatment hypothesis. Evidence for one application cannot automatically license another. This distinction allows computational advances to be evaluated without assigning them a treatment-selection role that their design cannot test.

## 2. Methods

### 2.1. Design and Interpretive Boundary

This critical narrative review combines a subtype-organized synthesis with structured quantitative appraisal; it is not a systematic review. The documented multi-source search was non-exhaustive, and full-text appraisal covered a purposively selected subset. Heterogeneous endpoints, cohorts, and validation designs precluded meaningful pooling of accuracy or direct ranking of models. Instead, we examined what each study validated, how it was evaluated, and which decision it could inform. The same predefined extraction and appraisal criteria were applied throughout. Proportions describe the appraised tier A set only, not the prevalence of practices across the field. The design supports within-set reporting patterns and statements about whether included studies meet specified readiness criteria. Selection and assessment limitations are discussed in Section 10.

### 2.2. Study Identification

We searched PubMed/PMC, Crossref, OpenAlex, Semantic Scholar, Unpaywall, bioRxiv/medRxiv, and arXiv, supplemented by citation tracking. The search covered English-language primary studies from January 2020 to 18 June 2026. Eligibility required AI or machine-learning prediction of breast-cancer neoadjuvant response with at least one performance metric. Screening or diagnosis outside this setting, segmentation or subtype classification alone, reviews, protocols, and animal or cell-only studies were excluded. Earlier landmark trials and guidelines served as background; preprints and trial-registry records supplied context rather than appraised outcome evidence.

**Figure 2:**
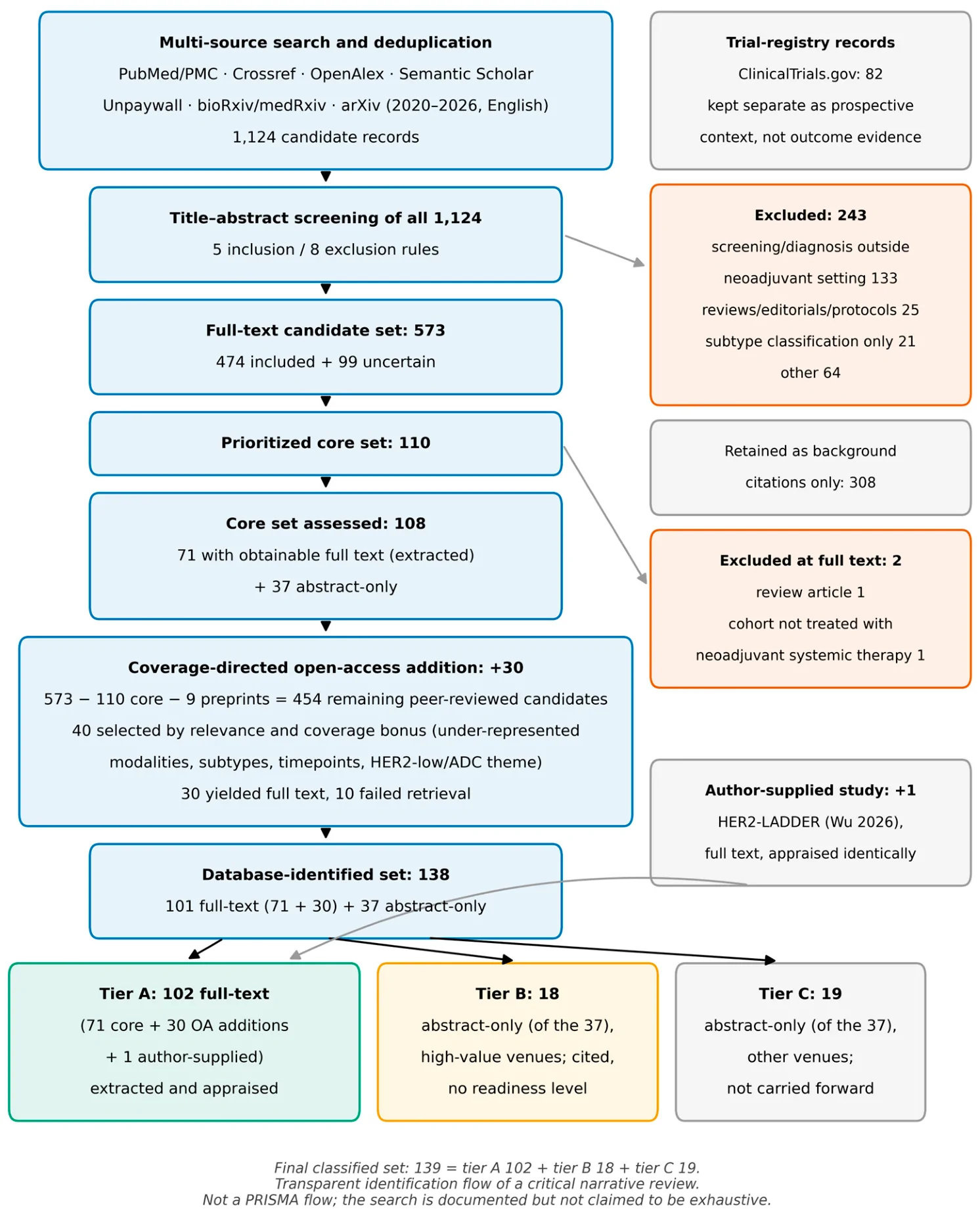
Study identification and tier classification. Multi-source search and deduplication yielded 1124 candidate records (January 2020 to 18 June 2026, English), with 82 trial-registry records held separate as prospective context. Title–abstract screening produced 474 included and 99 uncertain records, 243 exclusions with reasons, and 308 records retained only as background citations. From the full-text candidate set (*n* = 573), 110 records were prioritized as the core set; 2 were excluded at full-text review, leaving 108 (71 full-text, 37 abstract-only). A coverage-directed open-access addition then selected 40 records from the 454 remaining peer-reviewed candidates, of which 30 yielded full text, giving 138 database-identified records; one author-supplied study completed the set of 139. The final classification comprises tier A (102 full-text studies, extracted and appraised), tier B (18 abstract-only, cited without readiness grading), and tier C (19, not carried forward). The diagram documents a transparent narrative process; it is not a PRISMA flow, and the search is not claimed to be exhaustive [6].

Figure 2 documents identification, core prioritization, coverage-directed full-text additions, and final tier classification; Supplementary Methods S1 retains the complete numerical cascade and selection rules. Prioritization combined relevance with coverage of subtype, decision timepoint, modality, and treatment theme. Each study was classified by these dimensions. Pooled performance was not extrapolated to an individual subtype.

For corpus counts, a named subtype required a cohort restricted to that subtype by design. Mixed cohorts remained mixed even when one subtype predominated. For clinical synthesis, separately reported subtype results could inform the corresponding decision question. This distinction prevents assigning a pooled outcome to patients for whom performance was never estimated. Full-text prioritization sought coverage across these questions rather than statistical representativeness.

### 2.3. Evidence Tiers

Tier A comprised 102 full-text studies, including the author-supplied HER2-LADDER report [6], all extracted and appraised in Supplementary Table S1. Tier B comprised 18 abstract-only reports from high-value venues, cited as supporting evidence without readiness grading. Tier C comprised 19 remaining abstract-only records and was not carried forward. Tiers describe access to appraisal material, not scientific quality: a tier B study may be stronger than a tier A study. Venue-based prioritization and full-text accessibility therefore require the selection caveat in Section 10.

An abstract may establish that a model and a reported association exist, but usually cannot verify data splitting, calibration, or the independence of validation cohorts. Abstract-level citations therefore supplement clinical context without contributing to the quantitative appraisal. Conversely, obtaining a full text permits assessment without certifying methodological quality. Keeping these two judgments separate is necessary to interpret the evidence tiers correctly.

### 2.4. Appraisal and Clinical Readiness

Extraction covered cohort, subtype, regimen, endpoint, validation, sample size, and reported metrics. Risk of bias was assessed with PROBAST, referring to PROBAST+AI [7,8]. Reporting was read against TRIPOD+AI, CLAIM 2024, and CLEAR [9,10,11].

The author-defined L1–L5 ladder grades validation and translational maturity, not accuracy; it is not an externally validated standard. L1 denotes internal validation, including any random same-source split. L2 requires temporal or geographic separation from development. L3 requires frozen-model prospective observational or silent evaluation without altering care. L4 requires live workflow influence with measured impact. L5 requires prospective comparative model-guided intervention or regulated deployment with prospective outcome surveillance. High AUC, calibration, DCA, and reader comparisons cannot independently raise a level.

Supplementary Methods S2 preserves mandatory criteria, exclusions, promotion rules, validation subtypes, and the prior four-study re-audit. It also explains the distinction from bias appraisal and the relationship to DECIDE-AI [12]. All counts use the grading documented in Supplementary Methods S2.

## 3. Clinical Decisions and the Model Landscape

### 3.1. Decision Landscape

The relevant clinical decision determines what a response model must demonstrate. Pretreatment selection, on-treatment adjustment, and post-treatment management require different evidence, even within the same subtype. Figure 1 locates these decisions. A model can estimate the outcome under observed care while leaving the consequences of changing that care unanswered.

In HR+/HER2− disease, the pretreatment question is often whether chemotherapy is needed at all. Low pCR frequency and its weaker survival association limit the relevance of a pCR classifier to that choice. Endocrine sensitivity, residual burden, and recurrence risk are more closely aligned targets. During treatment, detecting endocrine futility would require longitudinal evidence early enough to guide a switch. After treatment, nodal response may inform surgical planning [13]. Recurrence and disease-free-survival models remain prognostic [14,15]; nodal-response predictors must also be compared with routine factors such as clinical response and Ki-67 [16].

In HER2+ disease, observed residual disease already supports post-neoadjuvant escalation. Pretreatment or early-response estimates instead raise de-escalation questions, including whether anthracyclines or part of chemotherapy could be omitted. The safety requirements differ because omission depends on an outcome that has not yet been observed. Multimodal, histopathological, radiomic, and clinicopathologic models predict fixed-regimen pCR [17,18,19,20]. Their discrimination cannot establish the residual-disease risk under a reduced regimen. The missing evidence concerns the proposed treatment change, even where prediction is externally validated.

In TNBC, pCR is more strongly associated with survival, but a high probability under immunochemotherapy does not show that immunotherapy or chemotherapy components are dispensable. Gene-expression and radiomics models provide subtype-restricted predictive evidence [21,22]. Stromal tumor-infiltrating lymphocytes (sTILs), assessable on routine hematoxylin-and-eosin (H&E) slides, provide a clinically accessible comparator. Complex models need to show useful increment beyond this baseline, particularly when proposing a drug-sparing decision.

Longitudinal monitoring spans all subtypes but has a separate evidentiary gap. External multicenter MRI validation, multi-timepoint multimodal prediction, and prospective longitudinal testing show that response estimates can be updated [23,24,25]. The additional information may identify eventual responders earlier. However, demonstrating that stopping or switching therapy at that time improves outcomes requires a model-guided strategy evaluation. Monitoring studies therefore contribute response evidence without establishing a safe adaptive treatment pathway.

### 3.2. Models, Endpoints, and Subtype Coverage

**Figure 3:**
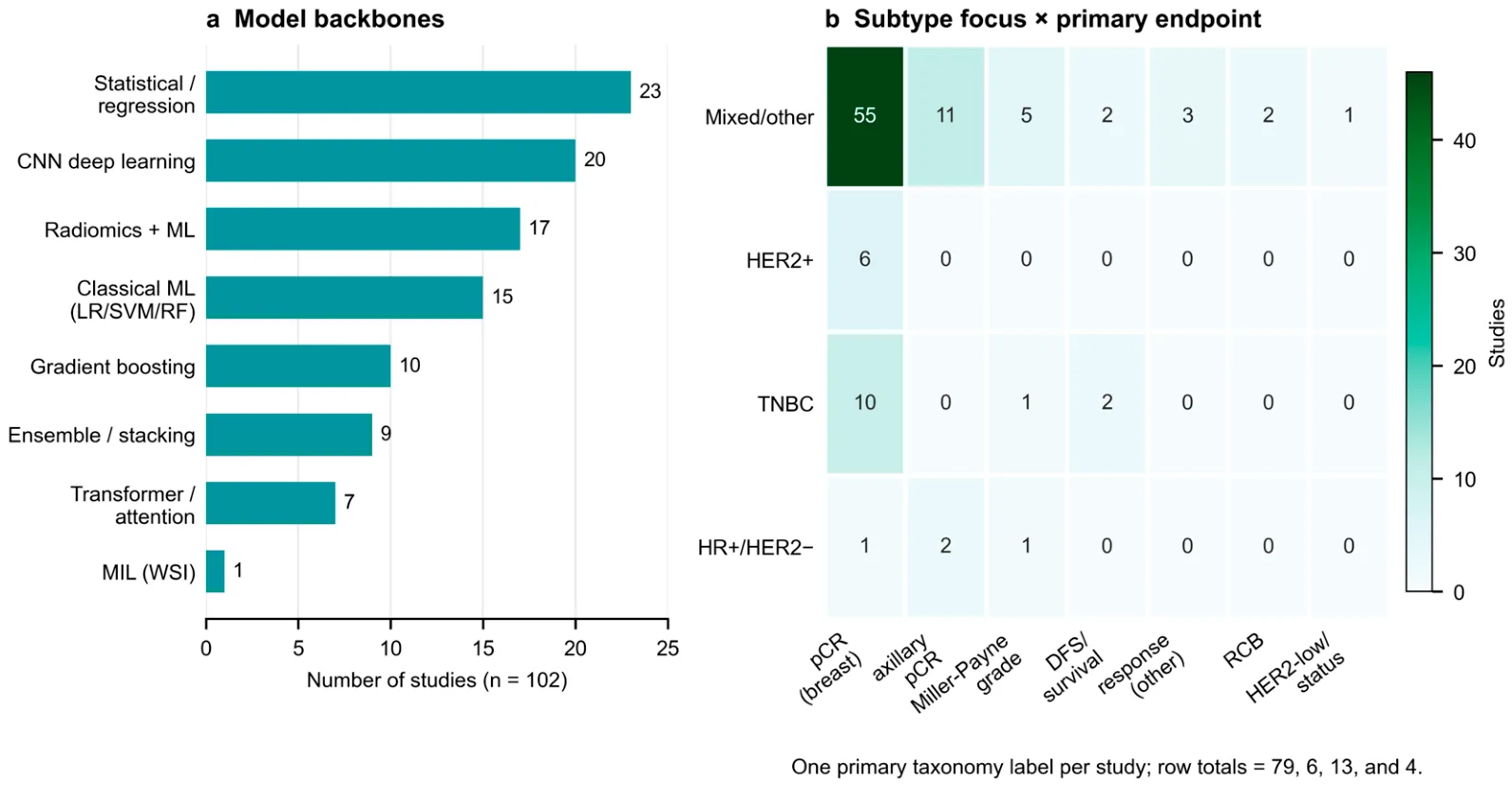
Model taxonomy across the 102 studies: (**a**) model backbones and (**b**) subtype focus by endpoint.

Model families largely follow the available data. Regression and classical machine learning use selected clinical, radiomic, or molecular features. Convolutional networks learn imaging representations, while transformers and attention mechanisms support spatial or longitudinal integration. Gradient boosting and stacking combine tabular or model-level information. Table 2 and Figure 3a summarize these families; Supplementary Note S3 retains the backbone census and parameterization detail.

The distribution in Figure 3b and Table 3 uses mutually exclusive design-based categories: 79 mixed/other studies, 6 HER2+-restricted, 13 TNBC-restricted, and 4 HR+/HER2−-restricted. TNBC-predominant cohorts enrolling other subtypes remain mixed/other. Dedicated subtype designs are uncommon, especially in HR+/HER2− disease. This partition differs from decision-cell mapping, where a mixed study can inform several cells through separately reported subtype results.

MRI is the most frequent single imaging source, and multimodal combinations draw on imaging, pathology, clinical variables, and omics. Recent work extends to spatial–semantic biopsy analysis, explainable multiomics, transcriptomic–histopathological integration, inferred spatial transcriptomics, and ultrasound pretraining [30,31,32,33,34]. These advances broaden representation learning; their clinical significance still depends on the outcome, validation setting, and comparator. Model capacity is therefore an appraisal consideration rather than a basis for ranking technologies. Learned features may improve discrimination while remaining difficult to audit across centers or scanners.

**Table 3:** Subtype-conditioned distribution of (a) imaging/data modalities, (b) evaluation endpoints, and (c) model backbones with clinical-readiness ceilings across the 102 tier A studies (study counts). Abbreviations: MRI, magnetic resonance imaging; PET, positron-emission tomography; DBT, digital breast tomosynthesis; pCR, pathologic complete response; RCB, residual cancer burden; MP, Miller–Payne; DFS, disease-free survival; HER2, human epidermal growth factor receptor 2; CNN, convolutional neural network; ML, machine learning; LR, logistic regression; SVM, support vector machine; RF, random forest; L1–L3, clinical-readiness levels.

**(a)** Subtype × modality																
**Subtype focus**	**multimodal**	**MRI**		**ultrasound**		**clinicopath**		**pathology**	**omics**	**PET**		**liquid biopsy**		**mammo-DBT**		**Total**
Mixed/other	24	21		12		10		5	2	3		2		0		79
HER2+	1	0		1		1		1	1	1		0		0		6
TNBC	4	3		0		1		3	2	0		0		0		13
HR+/HER2−	2	0		0		1		1	0	0		0		0		4
**(b)** Subtype × endpoint																
**Subtype focus**	**pCR (breast)**		**axillary node pCR**		**MP grade**		**DFS/surv**		**resp other**		**RCB**		**HER2 low**		**Total**	
Mixed/other	55		11		5		2		3		2		1		79	
HER2+	6		0		0		0		0		0		0		6	
TNBC	10		0		1		2		0		0		0		13	
HR+/HER2−	1		2		1		0		0		0		0		4	
**(c)** Subtype × backbone and clinical-readiness																
**Subtype focus**	**Studies**				**Dominant backbone(s)**				**Readiness (L1/L2/L3)**				**Ceiling**			
Mixed/other	79				CNN deep learning (17); Statistical/regression (16)				34/40/5				L3			
HER2+	6				Statistical/regression (3); CNN deep learning (2)				1/5/0				L2			
TNBC	13				Classical ML (6); Statistical/regression (3)				6/6/1				L3			
HR+/HER2−	4				No dominant family (one study each)				2/2/0				L2			

Notes: Modality and endpoint panels use one primary taxonomy label per study, so each row sums to its mutually exclusive study total. The separate subtype-by-timepoint evidence map presented later is non-exclusive. A study is assigned to a named subtype only when its design is restricted to that subtype; subtype-predominant mixed cohorts remain mixed/other. Readiness (L1/L2/L3) gives the count of studies at each level; ceiling is the highest level reached.

Endpoint variation is more consequential than architectural labels. The pCR definitions grouped in Table 1 differ in whether residual in situ disease or nodal involvement is allowed. Supplementary Table S2 retains the verbatim nine-category breakdown. These differences alter event frequency and the meaning of a positive prediction. Equal AUCs under different definitions do not imply equal predictive performance for the same clinical task. The distinction also affects threshold selection: a probability calibrated for one definition cannot simply be interpreted as the probability of another.

pCR and RCB answer related but distinct questions. pCR records absence of invasive residual disease as a binary event; RCB grades remaining disease on a continuous index with prognostic strata. Axillary pCR addresses nodal management, survival endpoints address longer-term prognosis, and immune or HER2 scores characterize tumor features. A model validated for one endpoint cannot be assumed to predict the others. Table 4 organizes primary endpoints and evaluation metrics without treating them as interchangeable.

**Table 4:** Evaluation endpoints, dominant metrics, and coverage of external validation, calibration, and decision-curve analysis across the 102 tier A studies, by primary modeled endpoint. Abbreviations: AUC, area under the receiver operating characteristic curve; CI, confidence interval; DCA, decision-curve analysis; pCR, pathologic complete response; RCB, residual cancer burden; TIL, tumor-infiltrating lymphocyte; DFS, disease-free survival; HER2, human epidermal growth factor receptor 2.

Evaluation Endpoint	Studies	Dominant Metric(s)	External Val. (Yes)	Calibration (Yes)	DCA (Yes)	Representative Study
pCR (breast)	72	AUC/ROC; sens/spec; accuracy	38	11	27	Sammut et al. [17]
Axillary/nodal pCR	13	AUC/ROC; sens/spec; accuracy	7	7	11	Li et al. [35]
Miller–Payne/ordinal grade	7	AUC/ROC; sens/spec; accuracy	1	0	4	Chen et al. [36]
Residual cancer burden (RCB)	2	AUC/ROC; sens/spec; accuracy	2	0	1	Li et al. [37]
TILs/immune spatial/other	3	AUC/ROC; accuracy; F1/κ	1	0	0	Peng et al. [38]
DFS/survival	4	AUC/ROC; C-index; sens/spec	3	0	1	Yu et al. [14]
HER2-low status	1	AUC/ROC; sens/spec; accuracy	0	1	0	Huang et al. [39]

**Notes:** “Studies” assigns each study once, by its primary modeled endpoint; the seven rows partition the 102 studies. Any-role endpoint tallies, in which one study can appear in several rows, are given in the lower section of Table 1. External validation, calibration, and DCA columns give the number of studies reporting each item. Totals: external validation, 52; calibration, 19 (fully reported); DCA, 44 (fully reported). **Abbreviations:** AUC, area under the receiver operating characteristic curve; ROC, receiver operating characteristic; sens/spec, sensitivity/specificity; DCA, decision-curve analysis; pCR, pathologic complete response; RCB, residual cancer burden; DFS, disease-free survival; HER2, human epidermal growth factor receptor 2; C-index, concordance index; F1, F1 score; κ, kappa statistic.

Evaluation concentrates on discrimination, whereas calibration and threshold-dependent net benefit are less completely reported. Section 8 quantifies these gaps. A comparison is interpretable only within its cohort, outcome definition, and validation design. Subtype-conditioned reporting should therefore test whether performance persists within the intended clinical population. Adding a subtype indicator to a pooled model does not by itself establish that evidence. The following sections assess each subtype against its decision, appropriate comparator, and remaining validation needs.

The study-level extraction also distinguishes a model’s primary target from outcomes reported in secondary analyses. For example, a paper may model breast pCR while additionally reporting nodal response or survival. Such reporting does not make those outcomes equivalent prediction tasks. The endpoint partition and broader reporting counts consequently answer different questions, as specified in the tables.

Within a backbone family, feature selection, dimensionality, and regularization further distinguish models. Statistical coefficients expose named predictors, whereas deep representations can encode imaging or spatial information without an equally direct interpretation. Those differences help explain what a model can reveal about response biology. They do not determine whether its output has been calibrated, independently tested, or compared with the current clinical decision rule. Supplementary Note S3 retains this computational detail for readers assessing individual pipelines.

## 4. HR-Positive/HER2-Negative Subtype

### 4.1. Clinical Questions and Appropriate Targets

Four questions structure this subtype: which tumors can avoid chemotherapy through endocrine sensitivity, what residual burden warrants escalation, when axillary surgery can be reduced, and whether futility can be detected early. The surgical question concerns treatment extent rather than systemic selection. Established benchmarks already include high-risk adjuvant CDK4/6 inhibition from monarchE and NATALEE [40,41], on-treatment Ki-67 from POETIC [42], and PEPI residual-risk assessment [43]. Observational comparisons of neoadjuvant and adjuvant chemotherapy remain confounded by baseline risk [44]. An AI model must add to these decision-relevant benchmarks. Table 5 summarizes the outstanding comparisons.

The dominant pCR target is often poorly matched to this biology. I-SPY 2 analyses and functional tumor volume prediction illustrate the limitations of a rare endpoint with weak surrogacy [49,60]. RCB, residual proliferation, and recurrence risk offer more defensible alternatives [37,50,51]. Subtype-stratified imaging and multimodal studies report pCR discrimination, but their heterogeneous outcomes and designs preclude a common performance ranking [23,45,46,47,48]. Supplementary Table S1 retains the individual metrics. Predicting response within a treated cohort also leaves unresolved whether chemotherapy should have been selected initially.

Evidence of incremental value remains limited. A radiomics nomogram incorporating Ki-67 improved on baseline imaging but not on an on-treatment signature, without an HR+/HER2−-specific increment [51]. A disease-free-survival nomogram showed net benefit over clinical staging, rather than over a genomic recurrence score [15]. Essentially no study compared AI head-to-head with clinically used recurrence scores. These comparator choices matter because outperforming staging or a weak imaging baseline does not demonstrate added value over the tools used for endocrine-versus-chemotherapy decisions.

Low event frequency also affects the clinical meaning of a positive result. Functional tumor volume modeling showed limited responder identification at a clinically constrained positive predictive value [49]. Improving a ranking statistic need not resolve that limitation. The relevant comparison asks whether AI changes a decision after the standard endocrine-sensitivity or recurrence-risk information is already available. That question remains more informative than another isolated pCR AUC in this subtype.

### 4.2. Residual Disease, Axillary Management, and Early Futility

Longitudinal imaging and integrated tissue-sequencing, circulating-tumor-DNA, and clinical models reached multicenter external validation for residual outcomes [37,50]. The imaging model reported DCA, but neither study established calibrated prospective deployment. Additional MRI-based RCB classification broadens residual-disease prediction [61]. This evidence supports continued validation of residual targets, without establishing which escalation strategy improves outcome for a predicted high-risk patient.

Axillary models in luminal disease report calibration and net benefit or validated nodal discrimination [52,62]. Conversely, low axillary-pCR frequency forced an FDG-PET/CT study to pool this subtype with others [63]. A mixed-cohort result cannot substitute for a subtype-specific surgical safety estimate. Predictive nodal evidence should also remain separate from endocrine sensitivity and chemotherapy benefit.

On-treatment imaging provides incremental response information but rarely validates an early stop-or-switch rule [37,49,51,64,65,66]. The three-gene endocrine-resistance score links cell models, retrospective survival cohorts, and eight post-letrozole samples; it remains exploratory [67]. Luminal phenotyping, subtype-specific ultrasound thresholds, and on-treatment MRI extend these signals [68,69,70]. A TNBC volume-reduction, sTIL, and Ki-67 model is an analogy rather than endocrine-futility validation [59].

### 4.3. Clinical Bottom Line: HR+/HER2−Disease

AI can stratify pCR or residual burden under observed treatment, with some residual-disease models reaching external validation. Clinically meaningful increment over Ki-67 and genomic recurrence tools remains unestablished. The evidence does not support choosing endocrine therapy over chemotherapy or stopping ineffective endocrine treatment on an AI score. These decisions need appropriately targeted and prospectively tested models.

The residual and axillary applications also differ in when their output becomes useful. A pretreatment estimate anticipates response, whereas a post-treatment nodal estimate addresses the extent of surgery after systemic therapy is complete. Combining these studies under a single maturity verdict would hide the different decisions they evaluate. The subtype summary therefore preserves their clinical separation.

## 5. HER2-Positive Subtype

HER2+ disease presents a clear distinction between escalation for observed residual disease and de-escalation based on predicted response. The KATHERINE pathway already supports post-neoadjuvant T-DM1, while anthracycline omission or shorter chemotherapy requires evidence of safety under the reduced regimen. Models must also demonstrate added value beyond routine hormone-receptor status and HER2 amplification. Table 5 locates these questions. A high pCR probability under dual blockade and chemotherapy does not answer what would happen if part of that treatment were removed.

MRI, ultrasound, elastography, deep learning, and digital-pathology studies report HER2+ performance, usually in subgroups of mixed cohorts [23,27,35,46,48,65,69,71,72,73,74,75,76]. HER2 scoring itself has also been linked to response [77]. Single-center optimism, falling external recall, differing clinical baselines, and reuse of public cohorts limit interpretation of apparent gains [17,20,64,78,79]. Individual AUCs remain in Supplementary Table S1 rather than being combined into a cross-study range.

HER2-LADDER derives cellular composition and spatial topology from baseline H&E and HER2 immunohistochemistry whole-slide images [6]. It combines cross-cohort validation, calibration, DCA, and spatial interpretability, making it an informative example of comparatively complete prediction reporting. However, its FASCINATE-N trial slides were assessed retrospectively. The temporal-validation AUC differs between reported locations, and the pCR label lacks a verbatim ypT0/ypN0 criterion. It remains L2 with high overall PROBAST risk, rather than evidence of prospectively successful treatment selection.

De-escalation evidence is still missing. A HER2DX-type gene-expression score was externally validated without calibration or DCA [53]; its probability threshold consequently lacks the evidence needed for a drug-omission decision. More fundamentally, fixed-regimen classifiers do not estimate the counterfactual response after anthracycline omission. ExteNET supplies clinical evidence for extended neratinib in the post-adjuvant setting [80], but the reviewed AI models do not predict differential T-DM1 or neratinib benefit. Observed residual disease, predicted residual disease, and predicted treatment benefit remain separate quantities.

Some models outperform clinicopathologic predictors or manual pathology reads [6,20,78]. HER2-LADDER also reports improved DCA relative to component models. These comparisons support incremental prediction within the evaluated cohorts. They do not establish superiority to externally developed systems, transportability to new practice settings, or prospective clinical utility.

The choice of comparator changes the claim that can be supported. An improvement over routine HR status or HER2 staining can demonstrate added predictive information. A comparison with manual pathology can support a reading aid. Neither evaluates whether the model-guided regimen is preferable to standard treatment. HER2-LADDER’s spatial explanations strengthen the interpretability of its response score, but a plausible explanation and a favorable decision curve still do not supply the missing strategy contrast.

### Early Response and the Clinical Bottom Line

Early-response studies include dual-tracer PET with internal cross-validation, temporally validated imaging fusion, and mixed-cohort longitudinal MRI [54,81,82]. Prospective enrollment alone does not raise an internally validated model above L1. A multimodal model reached L3 for axillary response, but that endpoint concerns nodal management rather than breast pCR or anthracycline omission [25]. No study linked an early AI-guided treatment reduction to comparative clinical outcomes.

HER2+ AI supports fixed-regimen response prediction, with some externally validated and calibrated models. It has not demonstrated that a predicted responder can safely receive less systemic treatment. Drug omission or shortening therefore requires strategy comparison and prospective evaluation of the proposed decision.

## 6. Triple-Negative Breast Cancer Subtype

For TNBC, response prediction is interpreted within chemotherapy and immune-checkpoint inhibition, while CREATE-X and OlympiA anchor post-neoadjuvant options for appropriate residual-disease populations [83,84]. Two questions govern AI’s contribution: whether it adds to standardized sTIL assessment and whether on-treatment change can identify immune non-response early enough for safe adjustment. Trial-embedded TIL comparison in HER2+ disease, TNBC immunochemotherapy histology, TIL-based prognosis, and longitudinal MRI provide relevant context [85,86,87,88]. The HER2+ comparison does not establish equivalent TNBC evidence. Table 5 summarizes the subtype’s predictive applications and limits.

### 6.1. Incremental Value over Stromal TILs

sTILs are an established, pathologist-readable marker with relatively low incremental cost when routine H&E slides and pathology infrastructure are available. The burden of proof therefore rests with the complex model. Added complexity needs to improve discrimination, calibration, or net benefit against a standardized sTIL read, rather than against random classification or a weaker clinical baseline.

Automated TIL scoring, spatial-graph features, transcriptomic nomograms, and biopsy models show heterogeneous predictive signals [55,57,89,90]. External TIL concordance declined in one study, and its spatial immune subtype was not an independent pCR predictor [55]. ARTEMIS provides a more direct incremental signal: tumor-volume reduction, sTILs, and Ki-67 improved discrimination over a clinicopathologic model [59]. However, validation used a random split within one institution and therefore remains L1. Calibration and DCA were absent, and treatment predated the immunotherapy standard. These limitations leave head-to-head value over standardized sTIL assessment unresolved.

### 6.2. Prediction and De-Escalation

Radiomics, longitudinal MRI, PET-genomics, post-treatment imaging, biology-informed models, and uncertainty-aware approaches extend pCR or residual-disease prediction [22,91,92,93,94,95]. Their positive predictions concern the regimen received. They do not estimate whether an anthracycline, platinum, or immunotherapy component could be removed without compromising outcome. ARTEMIS proposed identifying de-escalation candidates but acknowledged single-institution design, absent prospective validation, and pre-immunotherapy timing [59]. Disease-free-survival and metaplastic-TNBC prognostic models likewise stratify risk without identifying a safe treatment reduction [26,96].

This boundary remains relevant even when a model labels patients as potential de-escalation candidates. Candidate identification is a research hypothesis, with the associated safety threshold still to be tested. In TNBC, treatment context further matters because cohorts treated before routine immunotherapy do not directly validate predictions during contemporary immunochemotherapy. A model may retain prognostic information while requiring new validation for the treatment decision now being proposed.

### 6.3. On-Treatment Immune Non-Response

Longitudinal imaging gives the most mature on-treatment signal. A 3D convolutional model used baseline and cycle-four MRI in a blinded prospective TNBC test group, reaching L3 [56]. Its prospective AUC increased from 0.65 with baseline-only imaging to 0.83 with longitudinal input. This within-study comparison shows the information gained during treatment, but neither calibration nor DCA established a decision threshold. Volume-reduction models face similar limitations [59]. By the time a late-cycle signal is measured, the remaining opportunity for treatment adjustment also differs from a pretreatment prediction.

Peripheral-blood RNA-sequencing in I-SPY 2 linked early T-cell composite-score changes to TNBC response, with variable external performance attributed to batch, regimen, and overfitting effects [58]. Tryptophan metabolites, exosomal microRNAs, and myeloid-derived suppressor-cell profiles supply biological rationale rather than validated adjustment rules [97]. Molecular change is closely aligned with immune non-response, but its transportability remains fragile.

### 6.4. Clinical Bottom Line: TNBC

TNBC models provide response discrimination and an isolated prospective longitudinal signal. Increment beyond standardized sTIL assessment and the safety of drug-sparing decisions remain unproven. Detecting non-response and safely changing therapy must be evaluated as a linked clinical strategy before monitoring becomes treatment tailoring.

The comparator and timing questions must ultimately be addressed together. A delta signal that improves on baseline imaging may still add little after an on-treatment sTIL, volume, or proliferation assessment. The included studies rarely test this complete decision context. Their results justify targeted evaluation of monitoring strategies rather than assuming that additional measurements already identify a safe treatment adjustment.

## 7. Multimodal Fusion, Subtype Conditioning, and HER2-Low

### 7.1. Fusion Gains and Readiness

Multimodal fusion can improve discrimination within a study without advancing clinical readiness. Of 31 multimodal studies, 17 reached L1, 13 L2, and 1 L3; none reached workflow or interventional evaluation. Integrated clinical and molecular prediction and MuFi illustrate improvement over component inputs [17,46,98]. Further multiomics, MRI, PET, and radiopathomic studies report fusion gains, frequently without external testing [99,100,101,102,103,104,105,106]. Spatial graphs and longitudinal registration extend these approaches [107,108]; externally or temporally validated pathology, ultrasound, MRI, and TNBC studies broaden the evidence [19,22,48,73,109]. Related single- and multi-source approaches are retained for comparison [110,111,112,113,114,115,116,117].

The added inputs leave familiar appraisal problems. Calibration, Brier scores, and DCA are reported in selected studies, often on internal data [19,52,66,73,98,104,109,118]. Translation of modeled net benefit into absolute numbers remains uncommon [24,98]; it still estimates potential rather than observed treatment benefit. Increasing architectural complexity adds opportunities for overfitting and makes the complete pipeline harder to audit [24,27,46,119]. The prospective MIFAPS test followed model freeze, but the gap between training and external discrimination and remaining leakage concerns constrain interpretation [27]. Its prospective design advances validation, without proving utility from acting on the prediction.

Fusion comparisons are most informative when evaluated against the best single modality and a clinically credible baseline in the same patients. That design isolates the contribution of the added information without conflating it with a change in cohort or endpoint. The examples above support examining complementary inputs, but they should not be interpreted as a universal advantage for adding modalities. External performance of the final fused model matters even when individual components have already been validated.

### 7.2. Subtype Conditioning and Transportability

Including a binary HER2 variable in a pooled model does not establish subtype-specific performance. Stratified reporting and luminal-restricted designs provide more relevant evidence [48,64,66,68,73,101,102,120]. Yet performance can deteriorate after stratification: one pathology model reported external AUC 0.9005 for HER2+ disease and 0.5865 for TNBC [20]. Another declined from 0.84 in HR+/HER2− disease to 0.70 in TNBC [48]. These are within-study contrasts, not cross-study rankings. They show why a pooled multimodal AUC cannot support a subtype-specific treatment threshold.

Restricted designs reduce biological mixing but may narrow sample size and generalizability [22,52,54,68,92,121]. Reuse of I-SPY, DUKE, TransNEO, and TCGA also means different publications need not represent independent validation populations [64,66,99,122,123]. The required evidence is external discrimination, calibration, and net benefit within the intended subtype. Transportability should be judged for the complete pipeline and treatment context, not inferred from the name of a public dataset or a pretrained backbone.

This distinction is particularly relevant to a model that reports a high pooled AUC while subgroup estimates are sparse. The average may conceal different response frequencies or treatment contexts. Subtype conditioning must therefore be reflected in the target and evaluation, not only in an input column or subgroup label. A dedicated model and a pooled model with subtype-specific evaluation are different designs; neither are automatically sufficient for clinical tailoring.

### 7.3. Federated Learning and Foundation Models

No appraised study demonstrated clinical deployment supported by federated multicenter training. Foundation models supplied pretrained pathology features within supervised pipelines, as in MuFi and MIFAPS [27,46]; pretraining does not resolve their calibration, domain-shift, or prospective-utility gaps.

### 7.4. HER2-Low Scoring and ADC Response

DESTINY-Breast04 established the relevance of HER2-low status to trastuzumab deruxtecan (T-DXd) in its studied setting [124]. Within the reviewed neoadjuvant literature, MRI-based HER2-zero versus HER2-low classification and AI-TIL spatial scoring show early standardization signals [39,55]. The former lacks external validation and calibration; external concordance in the latter remains imperfect. These models address reproducibility of scoring, which is distinct from predicting benefit under a particular antibody–drug conjugate (ADC).

An ultrasound-radiomics model predicted response in HER2-low patients receiving conventional neoadjuvant chemotherapy [38]. It supplies no evidence about response to T-DXd. Prediction of changing HER2 status contributes a biological-evolution concept only [125]. Thus, AI shows preliminary HER2-low scoring signals, while no included study predicts response under an actual T-DXd neoadjuvant regimen. Scoring eligibility and predicting ADC benefit must remain separate claims.

## 8. Evidence Quality and Clinical Readiness

Table 6 summarizes appraisal under the rules of Section 2.4, with study-level evidence in Supplementary Table S1. The coverage includes MRI [36,126,127,128,129,130,131,132], ultrasound [133,134,135,136,137], pathology [29,138,139,140,141], PET and liquid biopsy [142,143,144], and clinicopathologic models [145,146,147,148,149,150,151].

**Figure 4:**
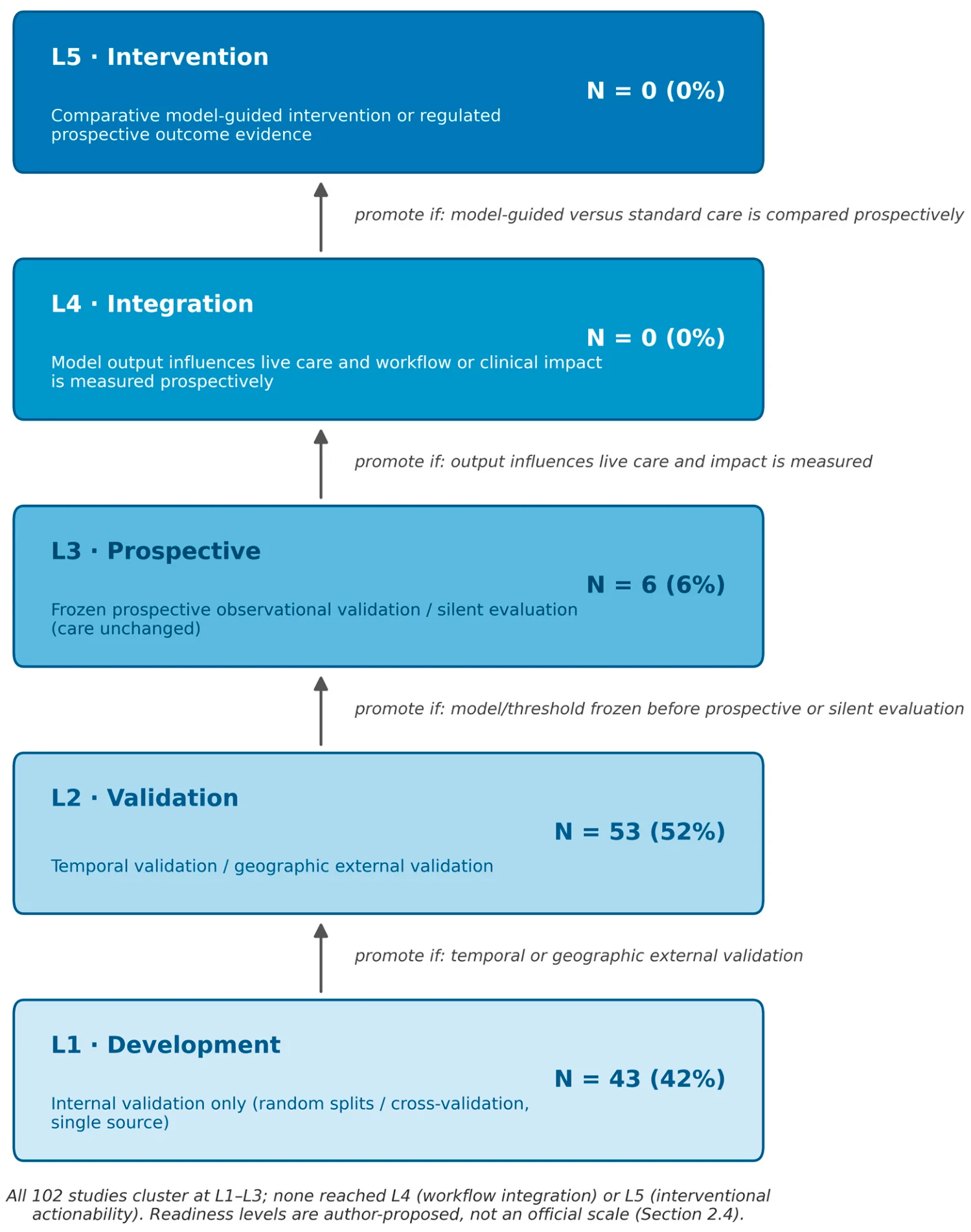
Author-proposed clinical-readiness ladder (L1–L5) across the 102 tier A studies, graded under the frozen L1–L5 rules of Section 2.4. All studies cluster at L1–L3 (L1 = 43, L2 = 53, L3 = 6); none reached L4 or L5.

Readiness remained concentrated at internal validation (L1, 43 studies) and temporal or geographic external validation (L2, 53). Six studies reached frozen prospective observational validation (L3); none reached L4 or L5. Figure 4 shows this distribution. These studies demonstrate prediction under observed treatment, without evidence that exposing clinicians to a model improves routine care. Supplementary Methods S2 distinguishes the validation designs grouped within each level. Readiness does not override risk of bias: an external test can coexist with weaknesses in model development or reporting.

**Figure 5:**
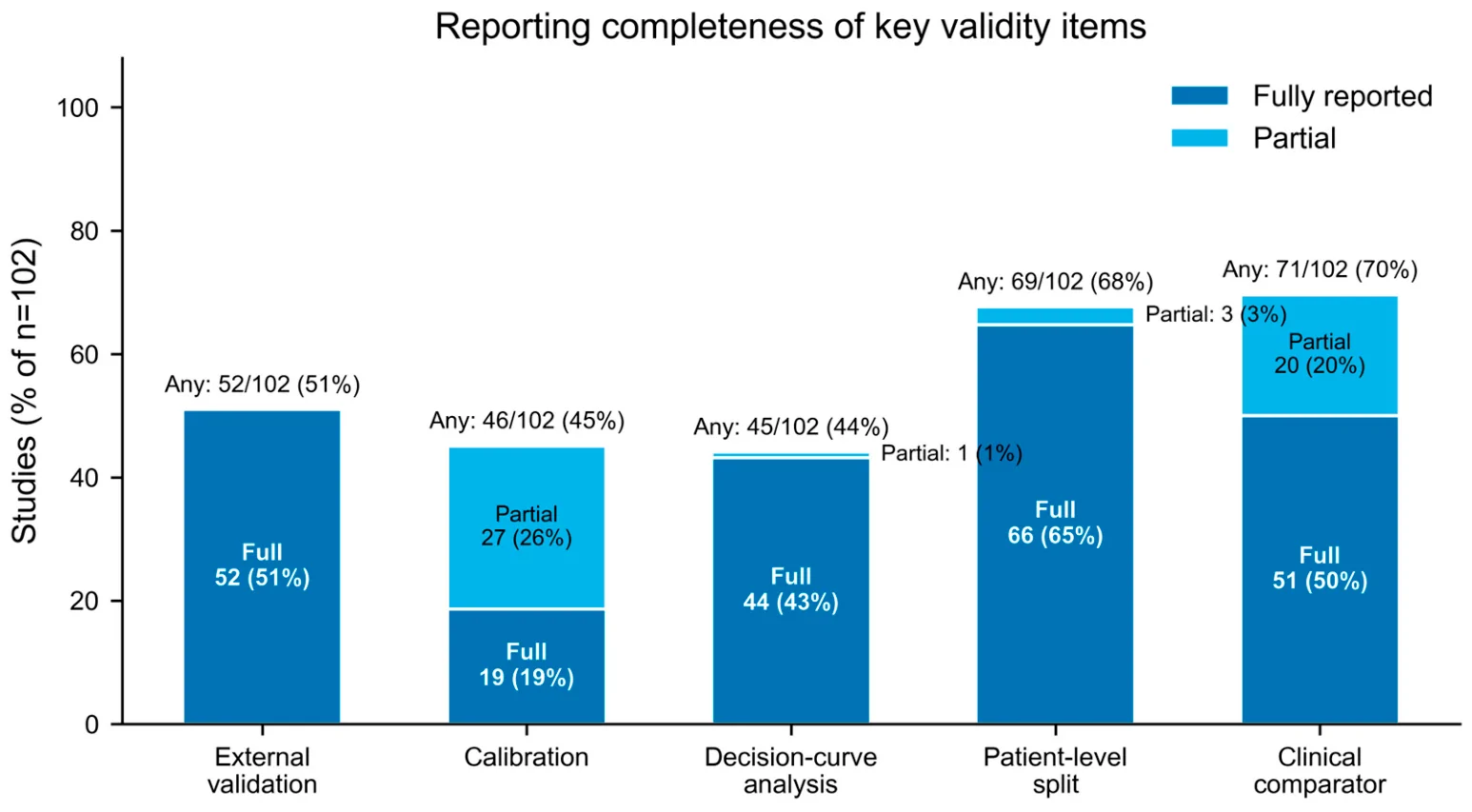
Reporting completeness of key validity items (*n* = 102). Dark segments show fully reported items, light segments show partially reported items, and labels above the bars show the cumulative number and percentage with any reporting. Full and partial categories are mutually exclusive.

Independent external validation was clearly reported in 52 of 102 studies (51%). Calibration was fully reported in 19 (19%) and partially in 27 more, totaling 46 (45%) with any reporting. DCA was fully reported in 44 (43%) and partially in one additional study. Figure 5 distinguishes full, partial, and cumulative reporting. Partial calibration reporting does not establish reliable probabilities. Patient-level separation was explicitly verified in 66 studies (65%); otherwise, slice- or lesion-level leakage could not be excluded from the report. Reporting flags and readiness use different criteria: the external-validation flag records a clearly reported item, whereas L2 also includes temporal separation.

**Figure 6:**
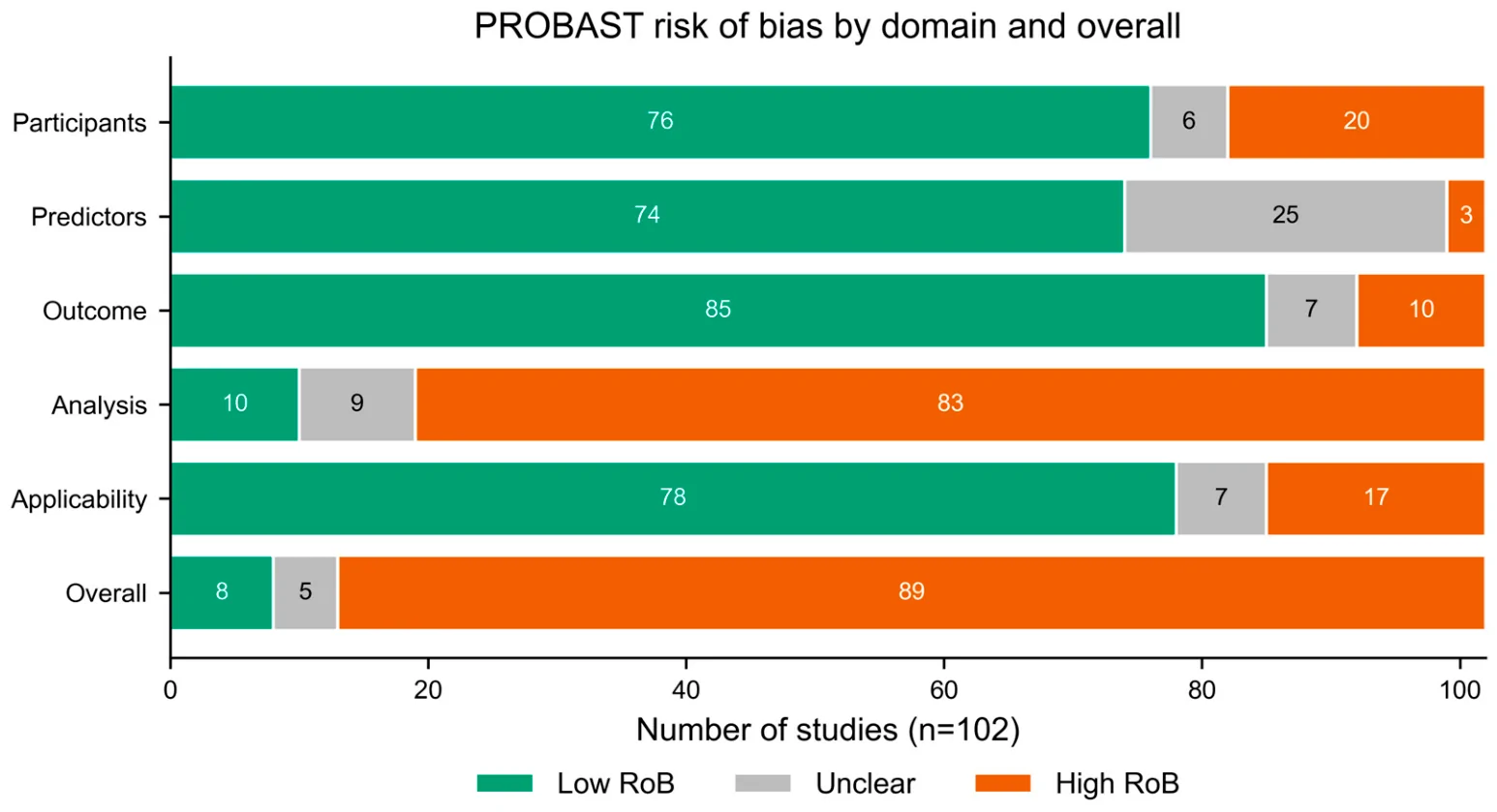
PROBAST risk of bias by domain and overall (*n* = 102). The analysis domain and overall rating are dominated by high risk of bias.

PROBAST rated 89 of 102 studies (87%) at high overall risk, 8 low, and 5 unclear. High risk concentrated in the analysis domain (83 studies), compared with participants (20), outcomes (10), and predictors (3). Figure 6 presents these domain ratings. Small event counts, absent sample-size justification, feature selection outside the training split, and full-sample normalization contributed to concerns about optimism. Leakage risk was high in 13% and moderate in a further 42%. A clinical baseline or clinician comparator was clearly reported in 51 studies (50%), but comparator strength varied.

**Figure 7:**
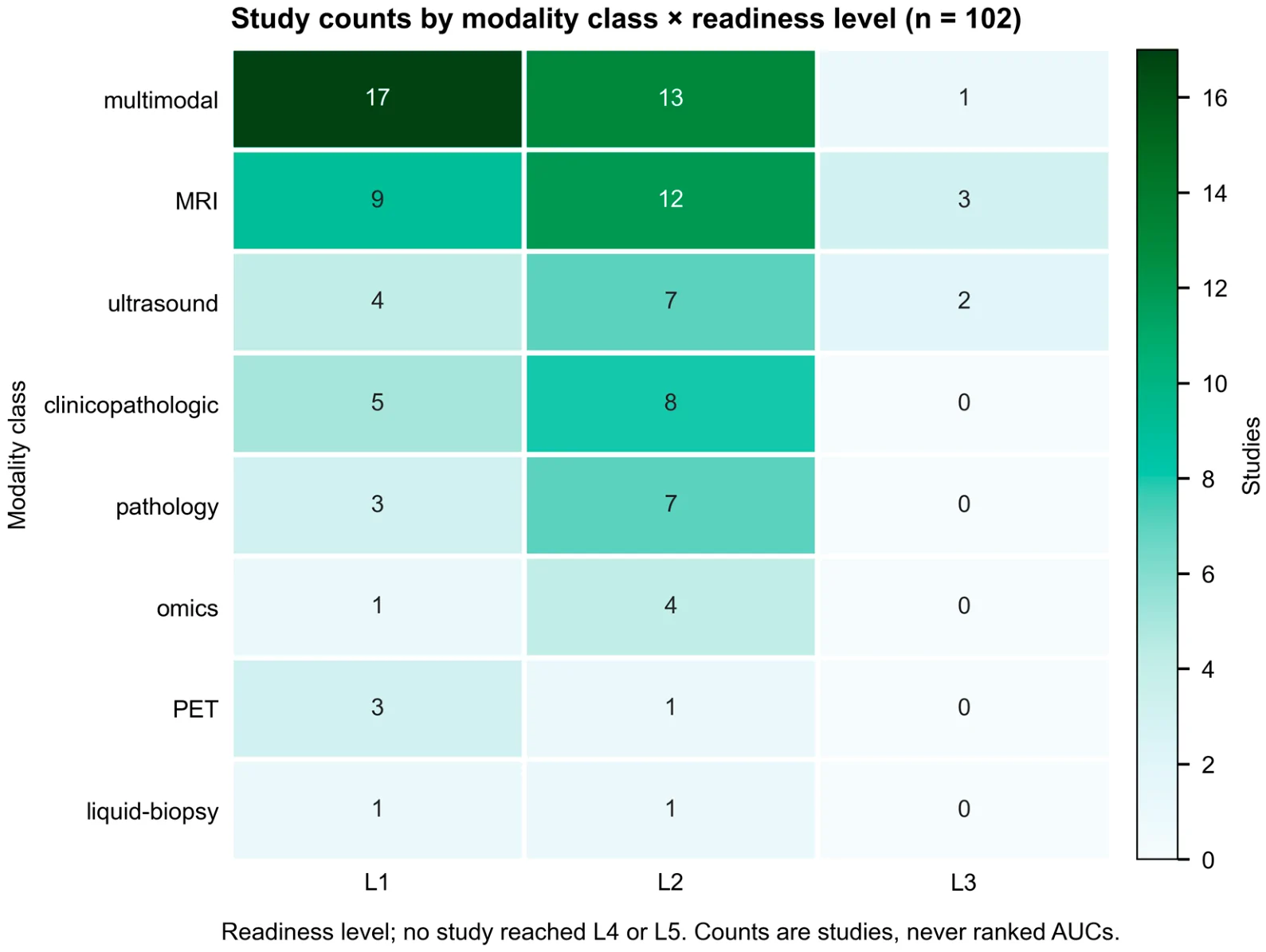
Study counts by modality class and readiness level (*n* = 102). All modalities cluster at L1–L3; none reached L4 or L5.

Architecture does not remove these limitations. Figure 7 shows that every modality remained within the lower readiness levels. Limited access to code, trained models, data, and preregistration also constrained reproducibility. Subtype-level calibration and decision thresholds are especially consequential when a model is proposed for treatment omission, because ranking patients cannot quantify the risk accepted by that decision.

Table 7 maps the resulting gaps by subtype and decision timepoint. Its cells are non-exclusive: mixed-cohort studies contribute only where relevant stratified evidence is available. Cell-level maturity summarizes the decision-specific evidence and should not be read as a new per-study grading. The concentration on retrospective response prediction and sparse on-treatment evidence explains why methodological diversification has not yet supplied treatment-selection evidence.

**Table 7:** Evidence map of subtype by decision timepoint across the 102 tier A studies. Each cell gives the number of studies informing that subtype and timepoint (*n*), the number with subtype-stratified results (stratified), and the imaging/data modalities represented. Abbreviations: HR, hormone receptor; HER2, human epidermal growth factor receptor 2; TNBC, triple-negative breast cancer; MRI, magnetic resonance imaging; PET, positron-emission tomography.

Subtype	Pretreatment	On-Treatment	Post-Treatment
HR+/HER2−	*n* = 48 (stratified 45); Modalities: MRI, PET, clinicopathologic, liquid-biopsy, multimodal, omics, pathology, ultrasound	*n* = 11 (stratified 11); Modalities: MRI, liquid-biopsy, multimodal, ultrasound	*n* = 22 (stratified 19); Modalities: MRI, clinicopathologic, liquid-biopsy, multimodal, omics, pathology, ultrasound
HER2+	*n* = 48 (stratified 42); Modalities: MRI, PET, clinicopathologic, multimodal, omics, pathology, ultrasound	*n* = 11 (stratified 9); Modalities: MRI, PET, multimodal, ultrasound	*n* = 18 (stratified 17); Modalities: MRI, clinicopathologic, multimodal, omics, pathology, ultrasound
TNBC	*n* = 56 (stratified 45); Modalities: MRI, PET, clinicopathologic, liquid-biopsy, multimodal, omics, pathology, ultrasound	*n* = 11 (stratified 10); Modalities: MRI, liquid-biopsy, multimodal, ultrasound	*n* = 22 (stratified 19); Modalities: MRI, clinicopathologic, liquid-biopsy, multimodal, omics, pathology, ultrasound

Note: A study informing more than one arm or timepoint is counted in each relevant cell, so cell counts sum to more than 102; this map is deliberately non-exclusive and must not be read as the mutually exclusive corpus distribution of Section 2.2.

Several quality dimensions can fail independently. Leakage inflates estimated performance; poor calibration undermines probabilities; weak comparators obscure incremental value; and absent workflow testing leaves consequences of use unknown. A study can address one of these problems without resolving the others. Reporting the dimensions separately prevents an externally validated label or a favorable AUC from standing in for a complete clinical assessment.

The practical reading of this appraisal is therefore decision-specific. A model proposed for risk stratification should establish reliable risk estimates in the intended population. A model proposed for treatment omission must additionally address the outcome under omission and the harm of a wrong decision. The latter claim cannot be inferred from the former, regardless of the model family. The following section brings these requirements together as the review’s clinically actionable conclusions.

## 9. From Prediction to Tailoring: What AI Can, Cannot, and Must Still Demonstrate

### 9.1. Where the Evidence Sits

The actionability ladder concerns the clinical claim, whereas the readiness ladder concerns validation and translation. Its first rung is predicting pCR or RCB under a fixed regimen. The second compares treatment strategies; the third estimates individualized treatment effects, including outcomes under alternatives. The fourth tests prospectively whether model-guided decisions improve outcomes. These distinctions prevent a prospective prediction cohort from being mistaken for a prospective treatment-selection trial.

The appraised response models remain on the first rung [6,24,25,27,28,35,56,58,152,153,154]. The prior appraisal found no study performing strategy comparison or ITE estimation. Cross-regimen validation of an I-SPY 2 peripheral-immune model tested prediction across settings rather than selection between treatments [58]. HER2-LADDER’s risk groups and retrospective trial-slide validation similarly do not provide a randomized strategy contrast [6]. Naming reduced, standard, or alternative regimens for predicted risk groups does not demonstrate their relative benefit for an individual.

Axillary prediction supplies the closest link to a changeable decision. FAIS-DL, dual-modality ultrasound, and prospective nodal cohorts report simulated reductions in unnecessary axillary lymph node dissection or improved false-negative performance [28,35,152]. These estimates concern surgical extent after fixed neoadjuvant treatment. They warrant prospective evaluation of surgical decisions, without establishing a basis for changing systemic drugs. Prospective pCR or nodal validation elsewhere retains the same distinction between observing predictions and testing model-guided care [25,27,56].

Thus, increasingly rigorous validation can strengthen a fixed-regimen predictor while leaving the treatment-selection question open. A clinically actionable conclusion should specify the decision that the evidence supports and the boundary it has not crossed. For this review, the practical boundary is between identifying likely response and demonstrating the consequences of acting differently because of that prediction.

### 9.2. What Models Can and Cannot Support

Current models can rank patients by the likelihood of pCR or residual burden under a specified regimen, sometimes with geographic external validation [23,73,78]. They can estimate axillary response for surgical research and refine response estimates when longitudinal imaging is available [28,35,56,152]. They also provide early assistance with inconsistently reproduced reads, including HER2-low and TIL assessment [55,85]. These are useful predictive or measurement tasks within the populations and conditions evaluated. Their usefulness should be judged against the existing clinical or pathology comparator for that task.

Current evidence cannot justify systemic drug omission, switching, escalation, or de-escalation on an AI output. Uncalibrated probabilities leave the risks at a proposed decision threshold uncertain. More fundamentally, fixed-regimen training cannot establish the outcome under an alternative regimen. Transportability across subtypes is incomplete, and the reviewed models lack measured live-workflow or interventional impact. Even a well-calibrated prediction needs additional causal and prospective evidence before it can support a treatment change.

The immediate clinical implication is to distinguish an investigational response estimate from a validated treatment rule. A research model can help define whom to study, which response features to measure, and which decision deserves prospective testing. It should not be described as established evidence for reducing or switching a drug solely because the predicted pCR probability is high. The proposed rule needs its own comparator, threshold, and outcome evaluation.

### 9.3. Evidence Required for Treatment Tailoring

First, validate the intended decision setting. Independent multicenter and multivendor evaluations should report discrimination, calibration curves and slopes, and DCA at clinically defensible thresholds within each subtype. Comparators should include the clinical, genomic, or pathology tools already used for the decision. Code, models, and compliantly shared data should permit appraisal of the entire pipeline under TRIPOD+AI, PROBAST, CLAIM, and CLEAR. Better reporting cannot remove bias, but it makes the proposed clinical use assessable.

Second, make on-treatment signals testable. Preregister longitudinal cohorts with fixed measurement times and harmonized delta-feature definitions. Evaluate whether change reflects biology rather than scanner or timepoint shift, and whether the information arrives early enough for the proposed adjustment. Early endocrine futility, HER2+ de-escalation, and TNBC immune non-response require distinct targets. Predicting eventual pCR should not substitute for evaluating the safety of the associated stop-or-switch rule.

Third, estimate outcomes under alternative treatments. Completed randomized neoadjuvant arms offer a source for strategy comparison and treatment-effect heterogeneity analysis. Candidate ITE models require validation of the treatment-effect estimates, not merely validation of outcome discrimination in each arm. HER2-low and ADC regimens are potential prospective testbeds, provided the outcome is response or benefit under the actual regimen being considered.

Fourth, evaluate the consequences of use. Following frozen prospective observational validation, live-workflow studies should measure how model output changes decisions and affects process, safety, or patient outcomes. Comparative interventional evaluation must then test model-guided against standard care, or appropriate regulated deployment must provide prospective outcome evidence. Retrospective reader comparisons and modeled net benefit cannot substitute for these steps. Federated learning and foundation models may help address data scarcity or domain shift, but their contribution must be demonstrated within this evaluation sequence.

These requirements are complementary. External calibration makes a score interpretable at a threshold; strategy comparison addresses the alternative treatment; workflow and intervention studies test what happens when the rule is used. Progress on one requirement does not eliminate the others. Designing studies around the intended treatment decision makes these dependencies explicit and keeps clinical evaluation aligned with the claim.

## 10. Ethics, Implementation, and Limitations

Clinical implementation requires fairness, interpretable outputs, regulatory assessment, and fit with existing care pathways [155]. Supportive care, including the scalp-cooling study, addresses tolerability rather than AI-based tumor response or systemic-treatment selection [156]. These patient-centered concerns should remain distinct from the decision a response model claims to inform.

Only 41 of 102 studies (40%) reported any subgroup performance. Aggregate AUC therefore cannot establish fair performance across age, race, ethnicity, scanner, institution, or subtype. Relevant populations need both discrimination and calibration assessed separately. Deployment also requires evaluation under applicable medical-device and change-control frameworks; no regulatory clearance was reported for the surveyed models. The absence of measured workflow impact prevents assuming that an accurate research score will improve care when presented to clinicians.

Interpretability remains largely post hoc, through saliency maps or feature attributions. Such displays can aid interrogation but do not establish causal correctness or safe treatment selection. Workflow evaluations should examine how users understand and act on an output, alongside its predictive accuracy. Standalone reader-performance gains leave these implementation questions unanswered.

The review’s own limitations constrain its conclusions. Its purposive, English-language narrative search and tier A full-text access favor particular venues and regions. All proportions describe the appraised set, not unbiased field-wide prevalence. Tier B reports were cited from abstracts and excluded from readiness grading. Screening, extraction, and appraisal followed one structured protocol with automated consistency checks and author adjudication, rather than duplicate independent assessment. Traceable records reduce but cannot eliminate misclassification. The readiness ladder is author-defined and has not been externally validated; Supplementary Methods S2 preserves its operational rules.

Most underlying studies are retrospective proof-of-concept reports, often with overlapping public data and optimism in performance estimation. The appraisal identifies limits of the included evidence, without asserting that no relevant unpublished or unselected study exists. Accordingly, the absence of workflow or interventional evidence applies to this appraised set. These boundaries remain essential when interpreting the review’s clinically focused conclusions.

## 11. Conclusions

Subtype-specific AI currently predicts response under observed neoadjuvant regimens more convincingly than it guides treatment selection. HR+/HER2− disease requires targets aligned with endocrine sensitivity and residual risk. HER2+ disease and TNBC offer stronger pCR-related signals, but a predicted response under treatment does not establish safety after reducing that treatment. Surgical-response models address a related decision and should be evaluated on their own terms.

The principal bottleneck is the evidence surrounding a prediction. Discrimination alone cannot establish reliable probabilities, transportability to the intended subtype, or benefit from acting at a particular threshold. Even prospective prediction validation leaves the counterfactual question unresolved when patients continue to receive the same care. Current findings therefore support further development and decision-specific validation, without supporting routine AI-guided changes to systemic drugs.

Progress requires independently validated and calibrated subtype-specific predictions, clinically timed longitudinal measurements, explicit strategy comparisons, and prospective testing of model-guided care. The treatment decision and acceptable risk should determine the model’s target, comparator, and evaluation design. This path preserves the value of response prediction while specifying what must be demonstrated before it can safely inform an individual patient’s treatment.

## Figures and Tables

**Table 1:** Endpoint definitions and their clinical meaning across the 102 tier A full-text studies. Abbreviations: pCR, pathologic complete response; DCIS, ductal carcinoma in situ; RCB, residual cancer burden; DFS/iDFS, disease-free/invasive disease-free survival; RFS, recurrence-free survival; OS, overall survival; EFS, event-free survival; DCA, decision-curve analysis.

Endpoint Category	Definition and Meaning	Studies	Decision Relevance
**pCR definition groups (*n* = 101 studies with a stated or inferable pCR label; full nine-category breakdown in Supplementary Table S2)**			
pCR—ypT0/is ypN0 (residual DCIS allowed)	No residual invasive cancer in breast and axillary nodes; residual DCIS permitted.	50	Most common and FDA-recognized pCR definition; the label most models are trained on.
Stricter or narrower variants	Strict ypT0 ypN0 (no residual in situ), breast-only ypT0/Tis, or standalone axillary pCR definitions.	4	Shift the event rate and the meaning of a positive prediction; not interchangeable with the standard label.
Operationalized through another scale	pCR equated to RCB-0, or represented by the top grade of a pathological-regression system (Miller–Payne, Sataloff, Japanese-society).	22	Ties the label to a different grading continuum; semantics are heterogeneous across systems.
Ambiguous or unverifiable	In situ status unstated, no verifiable verbatim definition, or other non-standard wording.	25	Without a verifiable label one cannot tell what the model predicts; flagged as non-comparable.
**Other endpoints and decision relevance (studies reporting each endpoint in any role, primary or secondary)**			
Residual cancer burden (RCB), continuous/graded	MD Anderson index grading residual disease RCB-0/I/II/III.	8	Tracks long-term outcome better than binary pCR and can inform escalation, but reported in a minority.
Axillary/nodal response (as primary or secondary endpoint)	Endpoint involves axillary nodal status (ypN0 conversion, axillary pCR).	22	Directly relevant to axillary surgical de-escalation (SLNB instead of ALND).
Radiological response (RECIST/radiological CR)	RECIST v1.1 or radiological complete response, usually secondary.	6	Imaging response is not pathological response; weak as a standalone decision basis.
Disease-/recurrence-free survival (DFS/iDFS)	DFS or iDFS, usually secondary/prognostic.	21	Direct long-term benefit measure; a few studies use it to test pCR surrogacy.
Recurrence-/relapse-free survival (RFS)	RFS (recurrence/relapse) used as an endpoint.	9	Close to DFS; reflects recurrence risk.
Overall survival (OS)	OS, usually secondary/exploratory.	8	The hardest benefit endpoint; rarely primary given limited follow-up.
Event-free survival (EFS)	EFS (incl. one study testing pCR as an EFS surrogate).	2	Key regulatory endpoint in neoadjuvant trials; very rare here.
Toxicity/adverse events/safety	Treatment toxicity or safety as a predicted endpoint.	0	Escalation trades off toxicity, yet no model addresses it—a key evidence gap.
Decision-curve analysis/net benefit (decision-relevance metric)	DCA/net benefit estimates net benefit under specified thresholds and assumptions; it is not an endpoint or prospective impact evidence.	44	Separates discrimination from possible threshold-dependent net benefit, but cannot replace prospective impact evaluation.

Notes: The pCR-definition rows classify the 101 studies with a stated or inferable pCR label; one study modeled a non-pCR endpoint only and is not assigned a category. The full nine-category breakdown of verbatim definition classes is given in Supplementary Table S2. Counts in the lower section tally any-role reporting (primary or secondary); a study reporting more than one endpoint appears in more than one row. AUCs obtained under different pCR definitions, cohorts, or subtypes are not directly comparable and must not be ranked.

**Table 2:** Model backbones and their typical parameterization across the 102 tier A studies, with the number of studies using each backbone by endpoint. Abbreviations: CNN, convolutional neural network; ML, machine learning; MIL, multiple-instance learning; pCR, pathologic complete response; RCB, residual cancer burden; MP, Miller–Payne; DFS, disease-free survival; HER2, human epidermal growth factor receptor 2.

Model Backbone	Typical Parameterization	Representative Study	pCR (Breast)	Axillary Node pCR	MP Grade	DFS/Surv	Resp Other	RCB	HER2 Low	Total
Statistical/regression	LASSO/elastic-net feature selection + logistic regression	Ni et al. 2025 [26]	15	4	1	2	1	0	0	23
CNN deep learning	ResNet/U-Net backbones; segmentation + deep features	Mao et al. 2025 [27]	14	3	3	0	0	0	0	20
Radiomics+ML	LASSO radiomic scores across imaging modalities	Yu et al. 2023 [14]	11	2	1	2	1	0	0	17
Classical ML (LR/SVM/RF)	RFE/LASSO selection + SVM/RF/naive Bayes	Chen et al. 2025 [28]	11	2	1	0	1	0	0	15
Gradient boosting (XGBoost/LightGBM)	tabular multimodal/multi-omic fusion	Huang et al. 2025 [23]	8	0	1	0	0	0	1	10
Ensemble/stacking	stacked base models across timepoints/modalities	Zhu et al. 2024 [25]	7	1	0	0	0	1	0	9
Transformer/attention DL	cross-modal/longitudinal attention fusion	Liu et al. 2025 [13]	6	1	0	0	0	0	0	7
Multiple-instance learning (WSI)	attention pooling over WSI patch embeddings	hou et al. 2026 [29]	0	0	0	0	0	1	0	1

Notes: Counts are study numbers; a study may use more than one endpoint, so row totals need not equal the study count. Backbone assignment reflects the primary modeling approach reported in each study.

**Table 5:** Subtype-specific AI: dominant modalities and model families, what the models demonstrably do, and distinctive insights. Abbreviations: MRI, magnetic resonance imaging; DCE, dynamic contrast-enhanced; DL, deep learning; ML, machine learning; WSI, whole-slide image; TIL, tumor-infiltrating lymphocyte; FTV, functional tumor volume; RCB, residual cancer burden; PPV, positive predictive value; ADC, antibody–drug conjugate; ITE, individualized treatment effect.

Subtype	Modalities & Model Families	What AI Demonstrably Does	Distinctive AI Insight
HR+/HER2−	MRI (incl. functional/DCE), ultrasound, clinicopathologic±genomic/hematologic, longitudinal imaging; radiomics+classical ML/nomograms, MRI deep learning, longitudinal delta models	Moderate pCR discrimination (subtype AUC ~0.78–0.86 [23,45,46,47,48]); FTV AUC 0.70 but only 20% sensitivity at PPV ≥ 50% [49]; shift to RCB endpoints [37,50,51]; an axillary-pCR nomogram with calibration+DCA [52]; recurrence/DFS stratification [14,15]	pCR is arguably the wrong target (rare ~20%, weak surrogate); the real value lies in RCB/residual, endocrine sensitivity, and whether AI beats the in-use Ki-67/genomic scores (essentially unconfirmed head-to-head); the low event rate suppresses PPV
HER2+	Digital-pathology WSI (H&E+HER2 IHC, spatial), MRI radiomics/DL, dual-tracer PET, clinicopathologic; gene-expression scores, multimodal fusion	HER2-LADDER [6], multi-cohort with calibration, DCA and spatial interpretability; gene-expression pCR score, external AUC 0.80/0.77 [53]; dual-tracer PET prospective cohort [54]; HER2-low AI-TIL scoring κ 0.68–0.72 [55] and MRI HER2-zero/low classification [39]	Closest an AI output comes to a drug decision, via HER2-low scoring for ADC/T-DXd eligibility. Fixed-regimen pCR ≠ de-escalation safety, and response under an actual T-DXd regimen remains unstudied
TNBC	Digital pathology (TIL/spatial immune, spatial graphs), MRI radiomics/DL with longitudinal delta, multi-omics/transcriptomics, liquid biopsy (RNA-seq, ctDNA), multimodal PET+genomics	The strongest predictive signal, with one prospective blinded test [56], cycle-4 AUC 0.83 vs. 0.65 baseline; AI-TILs κ 0.68–0.87 [55]; spatial-graph external AUC ~0.83 [57]; longitudinal T-cell composite [58]; TVR+sTIL+Ki-67 beats clinicopathologic-only [59]	Two open questions: whether AI beats the low-incremental-cost sTIL read, which no head-to-head study has tested, and whether longitudinal spatial or dynamic signals can monitor immune non-response. Predicting pCR ≠ safe to stop or reduce ICI
Cross-cutting: multimodal/subtype-conditioning	Cross-modal fusion (N = 31, the largest modality class); attention fusion, stacking ensembles, foundation models as frozen feature extractors	Fusion lifts discrimination, not readiness (of 31 studies: L1 = 17/L2 = 13/L3 = 1, none L4/L5); cross-subtype transfer collapses (HER2+ 0.90 → TNBC 0.59 [20])	Conditioning on subtype biology ≠ stacking modalities, and the conditioning is the hard part. Federated learning and foundation models remain proposals, not evidence

Notes: AUC values come from different cohorts, pCR definitions, and validation designs; they are for within-study reading only and must not be ranked across studies or subtypes (per-study values in Supplementary Table S1). Synthesis of the subtype sections and the multimodal section; citations are the manuscript’s existing reference numbers.

**Table 6:** Methodological quality and clinical readiness of the 102 tier A full-text studies, summarized by mutually exclusive subtype focus. Counts are numbers of studies. Per-study detail appears in Supplementary Table S1. Abbreviations: PROBAST, Prediction model Risk Of Bias ASsessment Tool; RoB, risk of bias; DCA, decision-curve analysis; L1–L3, clinical-readiness levels (L1 development/internal validation only; L2 temporal or geographic external validation; L3 frozen prospective observational or silent validation); TNBC, triple-negative breast cancer; HER2, human epidermal growth factor receptor 2; HR, hormone receptor.

Subtype Focus	Representative Studies	Studies (*n*)	PROBAST High RoB	Readiness L1/L2/L3	External Validation	Calibration	DCA	Clinical Comparator
Mixed/other	[25,136,152]	79	68	34/40/5	40	16	38	42
HER2+	[6,53,78]	6	5	1/5/0	4	2	2	4
TNBC	[22,55,91]	13	12	6/6/1	6	0	2	3
HR+/HER2−	[16,52,68]	4	4	2/2/0	2	1	2	2
All studies	—	102	89	43/53/6	52	19	44	51

Notes: A study is assigned to a named subtype only when its design is restricted to that subtype; TNBC-predominant mixed cohorts remain mixed/other. This rule differs from the non-exclusive subtype-cell map presented later in this section. PROBAST overall RoB: high 89, low 8, unclear 5. Readiness is an author-defined ladder, not an official scale; none reached L4 or L5. External validation, calibration, DCA, and clinical comparator columns count fully reported “yes” records; see Supplementary Table S1 for partial/unclear cases.

## Data Availability

No new patient-level data were generated in this review. The study-level extraction and appraisal data are available in Supplementary Tables S1 and S2.

## Supplementary Materials

The following supporting information can be downloaded at https://www.mdpi.com/article/10.3390/cancers18182947/s1. Table S1: Characteristics, per-study AI metrics, and PROBAST-based methodological appraisal of the 102 tier A full-text studies (spreadsheet); Table S2: Full breakdown of verbatim pCR-definition categories across the 102 tier A full-text studies; Supplementary Methods S1: Study identification and coverage-directed selection; Supplementary Methods S2: Full readiness rules and prior re-audit; Supplementary Note S3: Model-backbone census and parameterization.

## Abbreviations

The following abbreviations are used in this manuscript:

ADC	antibody–drug conjugate
AI	artificial intelligence
ALND	axillary lymph node dissection
AUC	area under the receiver operating characteristic curve
CLAIM	Checklist for Artificial Intelligence in Medical Imaging
CLEAR	CheckList for EvaluAtion of Radiomics research
CNN	convolutional neural network
ctDNA	circulating tumor DNA
DCA	decision-curve analysis
DCE-MRI	dynamic contrast-enhanced magnetic resonance imaging
DCIS	ductal carcinoma in situ
DFS/iDFS	disease-free/invasive disease-free survival
EFS	event-free survival
EPV	events per variable
FTV	functional tumor volume
H&E	hematoxylin and eosin
HER2	human epidermal growth factor receptor 2
HR	hormone receptor
ICI	immune-checkpoint inhibitor
IHC	immunohistochemistry
ITE	individualized treatment effect
MIL	multiple-instance learning
ML	machine learning
MRI	magnetic resonance imaging
NAC	neoadjuvant chemotherapy
OS	overall survival
pCR	pathologic complete response
PET	positron-emission tomography
PPV	positive predictive value
PROBAST	Prediction model Risk Of Bias ASsessment Tool
RCB	residual cancer burden
RECIST	Response Evaluation Criteria in Solid Tumors
RFS	recurrence-free survival
SaMD	software as a medical device
SLNB	sentinel lymph node biopsy
sTIL/TIL	stromal/tumor-infiltrating lymphocytes
SVM	support vector machine
T-DM1	trastuzumab emtansine
T-DXd	trastuzumab deruxtecan
TNBC	triple-negative breast cancer
TRIPOD+AI	Transparent Reporting of a multivariable prediction model for Individual Prognosis Or Diagnosis, AI extension
WSI	whole-slide image

## Appendix A. Glossary of Key Terms

Table A1 gives plain-language definitions of the technical terms used in this review, for readers less familiar with prediction-model methodology.

**Table A1:** Glossary of key terms used in this review.

Term	Plain-Language Definition
Discrimination	A model’s ability to rank patients, so that responders generally receive higher scores than non-responders; summarized by the AUC.
AUC (area under the ROC curve)	The probability that a randomly chosen responder receives a higher model score than a randomly chosen non-responder; 0.5 is chance, 1.0 is perfect ranking.
Calibration	Agreement between predicted and observed risk: when a calibrated model predicts a 30% chance of response, close to 30 of 100 such patients respond.
Decision-curve analysis (DCA)/net benefit	An analysis estimating potential net benefit at specified risk thresholds by comparing the model with treating all or no patients, under stated assumptions; it does not demonstrate prospective clinical benefit.
Clinical utility	Whether acting on a model’s output at a chosen risk threshold does more good than harm; assessed, but not proven, by decision-curve analysis.
Internal validation	Testing a model on data from its own development setting, such as cross-validation or a random split within one center.
Temporal validation	Testing on patients from the same center treated in a later period than the development cohort.
Geographic external validation	Testing on patients from centers that took no part in model development.
Prospective (observational) validation	Scoring a registered or prospectively enrolled cohort after the model is fixed, without the model altering care.
Individualized treatment effect (ITE)	The difference a given treatment would make for one particular patient, compared with the alternative.
Counterfactual evidence	Evidence about what would have happened under the treatment the patient did not receive; required for any claim that a model can select between treatments.
Strategy comparison	Evaluating a model’s predictions across two or more regimens, rather than under a single fixed regimen.
Multimodal fusion	Combining more than one data type, such as imaging with pathology or clinical variables, in a single model.
Radiomics	Extraction of large numbers of quantitative features from medical images for use in statistical or machine-learning models.
Deep learning	Machine learning built on multi-layer neural networks that learn features directly from raw data such as images, rather than from hand-engineered variables.
pCR (pathologic complete response)	No residual invasive cancer in the breast and axillary nodes at surgery; most commonly defined as ypT0/is ypN0.
RCB (residual cancer burden)	A validated continuous index grading residual disease from RCB-0 to RCB-III; finer-grained than the binary pCR label.
sTIL (stromal tumor-infiltrating lymphocytes)	A pathologist-readable immune marker in TNBC, prognostic and predictive, assessable on routine slides with relatively low incremental cost when tissue and pathology infrastructure are already available.
Clinical-readiness ladder (L1–L5)	The author-defined validation and translational-maturity scale used in this review: internal only (L1), temporal/geographic external validation (L2), frozen prospective observational or silent validation (L3), live workflow influence with measured impact (L4), and comparative model-guided intervention or regulated prospective outcome evidence (L5); defined in Section 2.4.
PROBAST	The Prediction model Risk Of Bias ASsessment Tool, which grades risk of bias in prediction-model studies across four domains.
TRIPOD+AI	The reporting standard for prediction-model studies involving machine learning.
Data leakage	Any construction that lets information from the evaluation data influence model development, such as feature selection performed outside the train–test split; it inflates reported performance.
Treatment tailoring	Changing an individual patient’s systemic therapy (omitting, switching, escalating, or de-escalating a drug) on the basis of a model output.
