# Supplementary material for: Beyond pCR Prediction: Subtype-Specific Artificial Intelligence for Treatment Tailoring in Breast Cancer Neoadjuvant Therapy
Source: Cancers (Basel). 2026 Sep 11;18(18):2947. doi: 10.3390/cancers18182947 (PMC13604375; doi:10.3390/cancers18182947)
Supplement: Supplementary file 1 [file cancers-18-02947-s001.zip › Supplementary Materials.pdf]

Supplementary Information

Beyond pCR Prediction: Subtype-Specific Artificial Intelligence for Treatment Tailoring in Breast Cancer Neoadjuvant Therapy

Supplementary Materials: The following supporting information is available in the online version. **Table S1**: Characteristics, per-study AI metrics, and PROBAST-based methodological appraisal of the 102 tier A full-text studies (separate spreadsheet file `Supplementary Table S1.xlsx`). **Table S2**: Full breakdown of verbatim pCR-definition categories across the 102 tier A full-text studies (typeset below). **Supplementary Methods S1**: Study identification and coverage-directed selection. **Supplementary Methods S2**: Full readiness rules and prior re-audit. **Supplementary Note S3**: Model-backbone census and parameterization.

Table S2: Full breakdown of verbatim pCR-definition categories across the 102 tier A full-text studies; condensed groups appear as Table 1 in the main text. Abbreviations as in Table 1.

**pCR definition category**	**Definition and meaning**	**Studies**	**Decision relevance**
pCR — ypT0/is ypN0 (residual DCIS allowed)	No residual invasive cancer in breast and axillary nodes; residual DCIS permitted (incl. 'irrespective of/including in situ', 'ypT0/Tis ypN0' equivalents).	50	Most common and FDA-recognized pCR definition; the label most models are trained on.
pCR — ypT0 ypN0 (stricter; in-situ must also be absent)	No residual invasive AND no residual in-situ disease in breast and nodes.	1	A stricter label lowers pCR rate and shifts the positive-class base rate; cross-study AUCs are not directly comparable.
pCR — 'no residual invasive cancer' (in-situ status unstated)	States only absence of invasive cancer in breast and nodes; whether residual DCIS is allowed is not specified.	5	Ambiguous wording; cannot be cleanly mapped to ypT0 vs ypT0/is, hurting label comparability.
pCR equated to RCB-0	pCR operationalized as a residual cancer burden index of 0 (RCB-0).	5	Ties pCR to the RCB continuum, enabling residual-disease gradation, though the pCR cut remains binary.
pCR via Miller-Payne / Sataloff / Japanese-society highest grade	pCR represented by the top grade of a pathological-regression system (e.g. Miller-Payne grade 5, Sataloff, Japanese-society grade).	17	Grading systems are not strictly equivalent to ypTNM pCR (e.g. MP5 is breast-focused); label semantics are heterogeneous.
Breast-only pCR (ypT0/Tis; nodes excluded)	pCR defined on the breast tumor only (ypT0/Tis); nodal status is not part of the definition.	1	Omitting the axilla can overstate 'complete response'; not interchangeable with standard pCR.
Axillary/nodal pCR (apCR; standalone endpoint)	Endpoint is absence of residual tumor in axillary nodes (no macro-/micro-metastases or ITCs); not a breast pCR.	2	Targets axillary downstaging / de-escalation of ALND; a different clinical question from breast pCR.
pCR not the modeled endpoint / no verbatim definition	pCR is not the primary modeled label, or no verifiable verbatim definition is provided in the full text.	17	Without a verifiable label one cannot tell what the model predicts; flagged as non-comparable.
Other / unclassified verbatim definitions	Individual verbatim definitions not fitting the above (e.g. contrastive 'non-complete pathological response' phrasing).	3	Reminds readers that 'pCR' is not a single standardized label.

[1] Rows classify the 101 studies with a stated or inferable pCR label; one study modeled a non-pCR endpoint only. Main-text Table 1 groups these nine categories into four.

Supplementary Methods S1. Study identification and coverage-directed selection

References and figure/table numbers in the following methods and note refer to the main manuscript. These additions preserve methodological detail moved from the previous manuscript; no additional analysis was conducted.

Figure 2 traces the flow from identification to final classification. Multi-source search and deduplication yielded 1,124 candidate records; 82 trial-registry records were kept separate throughout as context on ongoing prospective work, not as outcome evidence. Title-abstract screening of all 1,124 records against these criteria produced 474 included and 99 uncertain records, together with 308 records retained only as background citations. It excluded 243 records: screening or diagnosis outside the neoadjuvant setting (133), reviews, editorials, or protocols (25), subtype classification alone (21), and other reasons (64). The 573 included-or-uncertain records formed the full-text candidate set, from which 110 were prioritized as a core set. Prioritization combined relevance score with coverage across subtype, clinical timepoint, input modality, and treatment theme. Two core records were excluded on full-text review (one review article, one cohort not treated with neoadjuvant systemic therapy), leaving 108: 71 with obtainable full text, which were extracted, and 37 that remained abstract-only.

The 573 records included 9 preprints, so removing the 110 core records left 454 peer-reviewed non-core candidates. To widen full-text coverage, 40 further records were selected from these 454 candidates. Selection ranked them by the same prioritization score, with a coverage bonus for under-represented modalities (digital pathology, omics, liquid biopsy, PET) and the HER2-low/ADC theme. Open-access availability was then verified through Unpaywall. Of the 40, 30 yielded usable full text and were extracted; the other 10 failed full-text retrieval and were not added. This addition supplemented rather than replaced the abstract-only records. It gave a database-identified set of 138 records (101 full-text, 37 abstract-only), and with one author-supplied full-text study (main-text Section 2.3) the final classified set is 139. Preprints were cited with their status made explicit and used only for context.

Supplementary Methods S2. Full readiness rules and prior re-audit

The clinical-readiness ladder (levels L1–L5) is an author-defined appraisal framework constructed for this review; it is not a published or externally validated standard. Its levels grade validation and translation maturity, never discrimination. Each level has a mandatory criterion and an explicit exclusion. L1 requires model development with internal validation only, including cross-validation, bootstrapping, or any random split of one source. Calling a same-source hold-out set "independent" does not promote it. L2 requires evaluation on data separated from development by time or place: later patients at the same center, or patients from centers or cohorts that took no part in development. A random same-source split is excluded.

L3 requires a frozen model and decision threshold to be evaluated in a registered or prospectively enrolled observational cohort, or in silent live-workflow deployment, without model output altering care. Retrospective reuse of a prospective-trial dataset is excluded. L4 requires live workflow integration in which model output is shown to clinicians and can influence care, together with measured process, decision, safety, or patient impact. A retrospective or standalone reader-performance comparison is excluded. L5 requires prospective interventional comparison of model-guided care with usual care, or regulated deployment with prospective patient-outcome surveillance. Model accuracy, DCA, cost modeling, or regulatory discussion alone cannot qualify.

Promotion follows four rules. (1) A study receives the highest level whose mandatory criterion it demonstrably meets. (2) Evidence must be reported in the study itself; an ongoing or planned trial does not count. (3) Neither discrimination magnitude nor a deep-learning or multimodal label raises a level. (4) A compromised data split caps a study at L1. As a corollary, calibration, DCA, reader comparisons, cost-effectiveness modeling, and regulatory status are recorded separately unless the level-specific workflow or intervention criterion is also met.

Each level still groups designs of unequal strength, and L2 is deliberately broader than the other

levels; rather than collapse this range, we report it. Of the 53 L2 studies, 48 include geographic external validation and 5 rest on temporal validation alone. We name the validation types consistently throughout, because internal, temporal, geographic external, and prospective validation carry different implications for generalizability. All six L3 studies were rechecked for a frozen-model prospective test and retained as prospective observational validations; none is a silent deployment. Gao et al. [24] remained L2 because its multinational reader study and external cohorts were retrospective and outside a live clinical workflow.

During revision, all 102 gradings were re-audited against the full L1–L5 rules above. Four studies whose only "independent test set" was a random same-source split were reclassified from L2 to L1; every count in this manuscript reflects the re-audited grading. Conceptually, the ladder parallels staged evaluation frameworks for clinical AI, including the DECIDE-AI recommendations for early live clinical evaluation [12]. It was built for this appraisal, however, and its levels should be read as operational definitions rather than as an external standard. The ladder is also orthogonal to PROBAST and TRIPOD+AI: a study can be externally validated yet at high risk of bias, and the two axes are reported separately throughout.

Study-level reclassification record

The prior re-audit reclassified four studies from L2 to L1 because their held-out data were random subsets of the development source. This record preserves the adjudications rather than introducing a new analysis.

Gilad 2025 (extraction ID 74; [126]) used a same-source BMMR2/ACRIN 6698–I-SPY2 random hold-out of 74 patients. Comes 2021 (ID 174; [64]) used a 26-patient test subset of the same I-SPY1 source. Adrada 2025 (ID 182; [59]) used a single-institution 1:1 ARTEMIS split; a prospective data source did not make the model evaluation prospective. Sutton 2020 (ID 199; [129]) used a same-source 80/20 hold-out of 56 patients and reported no external validation. Each therefore remained internal validation under the frozen rule.

Supplementary Note S3. Model-backbone census and parameterization

Statistical and classical machine learning methods dominate numerically. Statistical or regression-based models appear in 23 studies; classical machine learning approaches such as logistic regression, support vector machines, and random forests account for 15 studies, together roughly 38 of the 102. These methods typically apply explicit feature selection, most often least absolute shrinkage and selection operator (LASSO), elastic-net, or recursive feature elimination (RFE), applied to radiomic, clinicopathologic, or multi-omic variables before fitting a simple classifier. Radiomics combined with machine learning comprises 17 studies. These often layer LASSO-derived radiomic scores from multiple imaging modalities, or combine subregional and peritumoral radiomic features with genomic and clinical data, including positron emission tomography/computed tomography (PET/CT) radiomics from tumor and reference lymph node.

Convolutional neural networks (CNNs) and transformer or attention-based deep learning together account for 27 studies and represent the methodological frontier. CNNs appear in 20 studies, typically using established backbones such as ResNet or U-Net for tumor segmentation and feature extraction, or ensembling separate branches for MRI, whole-slide image (WSI), and clinical data. Transformer and attention-based approaches comprise 7 studies and include shared-private transformers for longitudinal multiregional ultrasound, hierarchical vision transformers with radiology-guided co-attention fusing WSI and MRI, and large language model embeddings applied to pathology reports. Multiple-instance learning appears in 1 study, using attention-based pooling over foundation-model patch embeddings from WSI for residual cancer burden (RCB) prediction, and represents the least represented backbone despite its growing role in computational pathology.

Gradient boosting methods, including XGBoost and LightGBM, appear in 10 studies and are used

almost exclusively for tabular multimodal or multi-omic fusion, such as longitudinal spatial-habitat radiomics with clinical variables, or whole-exome and whole-genome bisulfite sequencing integrated with clinical covariates. Ensemble and stacking approaches comprise 9 studies, averaging classifiers across multi-omic feature sets or stacking radiomic and deep feature representations across pre-treatment, mid-treatment, and delta imaging timepoints. Architectural sophistication does not ensure generalizability; several transformer and attention-based studies report strong internal discrimination but lack external validation on multi-center cohorts. Backbone selection and endpoint assignment are summarized in Table 2 and Figure 3a.

Parameterization tracks the backbone rather than the subtype. Statistical and classical machine-learning models are the most transparent, carrying tens of engineered features and a low-parameter classifier whose coefficients remain inspectable. Radiomics pipelines expand the raw feature pool into the hundreds before shrinkage collapses it to a compact signature. Convolutional and transformer networks move the parameter count into the millions and shift the effort from feature selection to architecture and regularization, so their reported hyperparameters center on backbone depth, learning schedule, and fusion strategy rather than on named predictors. Gradient-boosting and stacking models sit between these poles, tuning tree depth and ensemble weights over tabular inputs. This gradient matters for appraisal: low-parameter models expose what they use and invite calibration, while high-capacity models buy discrimination at the cost of interpretability and, as noted, often without external validation.

Input modalities

Multimodal fusion is the dominant input category, appearing in 31 studies. Among unimodal sources, MRI leads with 24 studies, predominantly using dynamic contrast-enhanced MRI (DCE-MRI). Ultrasound and clinicopathologic variables each contribute 13 studies; digital pathology, 10; omics, 5; positron emission tomography (PET), 4; and liquid biopsy, 2; no study used mammography or digital breast tomosynthesis as its sole modality. Within the tier A set, DCE-MRI is the most frequent single imaging modality, multimodal combination exceeds any single-source approach, and digital pathology and omics contribute smaller but distinct bodies of work.
